# A One Health systematic review of diagnostic tools for *Echinococcus multilocularis* surveillance: Towards equity in global detection

**DOI:** 10.1016/j.fawpar.2019.e00048

**Published:** 2019-04-16

**Authors:** Janna M. Schurer, Arlene Nishimwe, Dieudonne Hakizimana, Huan Li, Yu Huang, Jean Pierre Musabyimana, Eugene Tuyishime, Lauren E. MacDonald

**Affiliations:** a Cummings School of Veterinary Medicine at Tufts University, 200 Westboro Rd, North Grafton 01536, USA; b University of Global Health Equity, KG 7 Ave, 5th Floor, PO Box 6955, Kigali, Rwanda; c College of Veterinary Medicine, China Agricultural University, Beijing 100193, China; d HealthConnect Global Consulting, Hamilton L8L 7N3, Canada

**Keywords:** *Echinococcus_multilocularis*, Diagnostic_evaluation, Surveillance, One_Health, Systematic_Review

## Abstract

*Echinococcus multilocularis* is a zoonotic cestode of canid definitive hosts that is emerging as a parasite of medical and veterinary concern in regions of North America, Europe and Asia. Infection with the metacestode stage (alveolar echinococcosis – AE) is life-threatening, especially for patients who reside in low resource countries and lack access to modern diagnostic tests and treatments. The overall objectives of this One Health review were to systematically describe the diagnostic tests currently employed in endemic countries to detect *E. multilocularis* in people, canids and the environment, and to report the test characteristics of new diagnostic techniques for population surveillance. In this systematic review of English and Chinese language databases, we identified 92 primary records of *E. multilocularis* surveillance in canids (N = 75), humans (N = 20) and/or the environment (food, soil; N = 3) and 12 grey literature records that reported *E. multilocularis* surveillance or health systems protocols between 2008 and 2018. Surveillance for *E. multilocularis* was conducted using a broad range of combined morphological, molecular, immunological and imaging techniques. Nine studies reporting diagnostic evaluations for cestode or metacestode detection were identified, including studies on copro-antigen ELISA, copro-PCR, intestinal examination, Western Blot, magnetic capture RT-PCR and immunochromatography. Our dataset includes prevalence estimates for *E. multilocularis* in canids, people, or environment in 27 of the 43 endemic countries and reports data gaps in surveillance, laboratory methods, and diagnostic sensitivity. International consensus on gold standard diagnostic techniques and harmonization of human, canid and environmental surveillance data across political boundaries are needed to comprehensively assess the global burden and distribution of this parasite.

## Highlights

• Reports of AE prevalence were found most often from China, France, Poland and Canada.
• Accuracy of new molecular, immunological, and post-mortem tests is described.
• Surveillance across political boundaries and hosts is needed to estimate true burden.

## 1 Introduction

Alveolar echinococcosis (AE) is a debilitating medical condition that affects people and animals infected with the metacestode stage of *Echinococcus multilocularis* (Eckert et al., 2001). Such hosts are infected when they accidentally ingest cestode eggs that are shed into the environment in canid or felid fecal matter, contaminating soil, plants, and water. Definitive canid hosts are most commonly associated with human infections, namely, foxes (*Vulpes vulpes, V. lagopus, V. ferrilata*), wolves (*Canis lupus*), coyotes (*Canis latrans*), raccoon dogs (*Nyctereutes procyonoides*) and domestic dogs (*Canis familiaris*) (Deplazes et al., 2017). In the sylvatic lifecycle, rodents such as voles (e.g. *Arvicola* and *Microtus* spp.) are the primary intermediate hosts; however, AE is also reported in aberrant hosts such as domestic dogs, nonhuman primates, and swine (Deplazes et al., 2017).

Each year, *E. multilocularis* infects 11,400 to 29,600 new people, causes approximately 17,000 deaths, and incurs a global burden of 409,000 to 1.1 million Disability Adjusted Life Years (DALYs; Torgerson et al., 2015). For people and animals infected with AE, infections are characterized by multi-chambered cysts growing in liver tissue, with cysts occasionally expanding to other organs (Kern et al., 2017). Patients are often asymptomatic for years following infection, and eventually experience signs and symptoms related to the impaired function and eventual failure of the liver and affected tissues (Kern et al., 2017). The clinical outcomes for AE patients depend on cyst characteristics and immune status of the host, but especially on prompt diagnosis and access to modern treatment. If untreated, 90–100% of reported human patients die within 15 years of infection (Ammann and Eckert, 1996), and for that reason, AE continues to be a life-threatening condition for patients in low income endemic regions where medical access is limited.

*Echinococcus multilocularis* is geographically restricted to the northern hemisphere, but is widely distributed across countries in North America, Europe and Asia. The vast majority of human cases are reported from rural areas of western China (91%), followed by Russia (6%) (Torgerson et al., 2010). In focal regions of each of the three endemic continents, *E. multilocularis* is considered an emerging public health concern due to high prevalence in wild canids, detection of infected canids in new geographic areas, increased reports of AE in aberrant hosts (e.g. dogs, nonhuman primates), or increased incidence in human populations (Altintas, 2008; Romig et al., 2006; Schurer et al., 2015; Davidson et al., 2016). However, it is not always clear whether *E. multilocularis* is truly emerging or whether increased reports are the result of enhanced surveillance efforts and/or improved diagnostic techniques. In some regions, it is impossible to accurately characterize the burden of *E. multilocularis*, due to the unknown level of under-diagnosis, mis-diagnosis and under-reporting. These gaps can be partly attributed to the long interval between infection and disease onset, shortages of trained healthcare professionals, poor access to health services, lack of diagnostic tests, gaps in human and canid surveillance and lack of reporting infrastructure (EFSA and ECDC, 2016). Many endemic countries do not classify AE as nationally notifiable for people or animals (EFSA Panel on Animal Health and Welfare, 2015). Furthermore, human echinococcosis cases are often not identified to species level (Schurer et al., 2015; Piseddu et al., 2017), causing challenges in regions that are co-endemic for cystic and alveolar echinococcosis (Kern et al., 2017).

A wide array of morphological, molecular, immunological, and imaging tests exist for detecting *E. multilocularis* in people, canids, and the environment. These tests vary in diagnostic accuracy, cost, and resource requirements, such as skilled technicians, laboratory or diagnostic equipment, and reagent access (Conraths and Deplazes, 2015; Siles-Lucas et al., 2017). There is a recognized need to harmonize diagnostic strategies within the veterinary and medical communities to improve epidemiological data and to characterize regions of potential emergence (Conraths and Deplazes, 2015). There is also a need to improve equitable access of AE patients to state-of-the-art diagnostics and treatments currently unavailable in many endemic regions. Previous systematic reviews on this topic have been limited by geographic region or host species. Therefore, the objectives of this One Health study were (i) to systematically describe the current methods reported for population level detection of *E. multilocularis* in people, canids, and the environment in endemic countries, and (ii) to report diagnostic test characteristics and resource requirements of novel techniques evaluated against a gold standard at the population level.

## 2 Methods

This systematic review was conducted and reported according to the established guidelines “Preferred Reporting Items for Systematic Reviews and Meta-Analyses: The PRISMA Statement” (Moher et al., 2009). The protocol for the review is available on Prospero (registration #: CRD42018108935). Before starting, all reviewers received training in database searches, study selection, data extraction, and quality assessment to ensure uniformity within the research team. Ethics approval was not needed as this is a secondary literature-based study.

### 2.1 Search strategy

One author (JS) searched three English language databases (Embase, PubMed, Google Scholar) from January 1, 2008 up to and including Sept. 3rd, 2018. The following search strings were used:

PubMed: (“*Echinococcus multilocularis*” OR “*E. multilocularis*” OR “Alveolar echinococcosis”) AND ((“diagnosis” OR “diagnostic” OR “diagnos*” OR “test” OR “screen*” OR “detect*”) OR (“monitor*” OR “surveillance” OR “epidemiolog*” OR “prevalence” OR “burden”)).

Embase: ((“alveolar echinococcosis” or “*Echinococcus multilocularis*” or “*E. multilocularis*”).ab. or (“alveolar echinococcosis” or “*Echinococcus multilocularis*” or “*E. multilocularis*”).ti.) and ((diagnosis or diagnostic or diagnos* or test or screen* or detect* or monitor* or surveillance or epidemiolog* or prevalence or burden).ab. or (diagnosis or diagnostic or diagnos* or test or screen* or detect* or monitor* or surveillance or epidemiolog* or prevalence or burden).ti.)

Google Scholar: With all of the words: “*Echinococcus multilocularis*” OR “*E. multilocularis*” OR “Alveolar echinococcosis”; with at least one of the words: “diagnosis” OR “diagnostic” OR “test” OR “screen” OR “detect” OR “monitor” OR “surveillance” OR “epidemiology” OR “prevalence” OR “burden”.

Searches of Google Scholar excluded patents but included citations. One author (JS) reviewed the first 10 pages of results, and each page thereafter containing at least one relevant citation.

To capture Chinese-language publications, two co-authors (YH, HL) searched the China National Knowledge Infrastructure (CNKI) database on November 1st, 2018 using the following terms:

“多房棘球绦虫” 和诊断, “多房棘球绦虫” 和检测以及 “多房棘球绦虫” 和监测

English translation: (“*Echinococcus multilocularis*” AND diagnosis) OR (“*Echinococcus multilocularis*” AND test) OR (“*Echinococcus multilocularis*” AND surveillance).

One author (JS) searched multiple databases (GreyLit.org, OpenGrey, Science.gov, European Centres for Disease Control (ECDC), European Food and Safety Authority (EFSA), Government of Canada) for grey literature published from January 1st, 2008 up to October 14th, 2018 using the terms “*Echinococcus multilocularis*” OR “*E. multilocularis*” OR “Alveolar echinococcosis”. Using the same terms, a second author (LM) searched The University of Toronto's custom Canadian government document search engine (available at: https://cse.google.com/cse?q=+&cx=007843865286850066037:3ajwn2jlweq) for relevant Canadian federal, provincial and municipal documents published from January 1st, 2008 up to October 14th, 2018. We were unable to access the search databases of relevant Chinese grey sources (National Institute of Parasitic Diseases, China Ministry of Health, National Health Commission).

Peer-reviewed and grey literature searches were not restricted by language. All collected studies were collated in Zotero reference manager (https://www.zotero.org/) and duplicates were removed according to title and author.

### 2.2 Study selection

Primary studies were included if they described diagnostic methods used for *E. multilocularis* surveillance and/or diagnostic method evaluation. Institutional laboratory or surveillance protocols and institutional surveillance reports outlining prevalence of *E. multilocularis* were also included. Reference lists of relevant grey literature were searched for additional sources. In addition, as part of our One Health approach, inclusion criteria selected studies that reported outcomes from humans, canids (i.e. wolves, foxes, coyotes, raccoon dogs and domestic dogs), and/or the environment (e.g. soil, plants, food, water). We excluded data related to non-canid definitive and aberrant hosts as population level surveillance is uncommon for these animals, and we excluded data related to intermediate hosts as post-mortem examination is a widely used and accepted method of diagnosis. We also excluded studies where diagnostic method efficacy was conducted using an early stage index test in Phase I or Phase II of evaluation (Boelaert et al., 2018). This review focused on articles published between 2008 and 2018 because we wanted to capture and report the diagnostic methods currently being used for surveillance, and because the accuracy of older diagnostic tests has been reported elsewhere. Further, studies must have reported on detection of *E. multilocularis* within an endemic country. Two global reviews of AE prevalence and distribution were completed in the last 10 years, and we included countries listed as endemic by these publications to create a list of countries to be included in this review (Torgerson et al., 2010; Deplazes et al., 2017). When discrepancies occurred between the two reports, we considered whether a country shared a border with an endemic country, whether autochthonous animal or human cases had been recently reported, and whether the authors had found reliable secondary sources on which to base their decision. The 43 countries that we considered endemic for AE are listed in Table 1.

**Table 1:** Countries considered endemic for *Echinococcus multilocularis* for the purposes of this systematic review (n = 43).

Asia (N = 13)		
Azerbaijan	Kyrgyzstan	Tadjikistan
China	Japan	Turkey
Georgia	Mongolia	Uzbekistan
Iran	Russia^a^	Turkmenistan
Kazakhstan		

Europe (N = 28)		
Austria	Germany	Romania
Belgium	Hungary	Russia^a^
Belarus	Italy	Serbia
Bulgaria	Latvia	Slovakia
Croatia	Lithuania	Slovenia
Czech Republic	Luxembourg	Sweden
Denmark	Moldova	Switzerland
Estonia	Norway (Svalbard Island only)	The Netherlands
France	Poland	Ukraine
Fürstentum Lichtenstein		

North America (N = 2)		
Canada	United States of America	

a Note: Russia listed on 2 continents.

Case reports, case series, short reports, short communications, letters, conference abstracts, experimental infections, reviews or meta-analyses were excluded, as were studies that did not report the diagnostic test used. Finally, article selection was carried out using the following steps: (1) title and abstracts of all collected studies were screened for relevance according to the stated inclusion/exclusion criteria; (2) articles that were deemed to be relevant, or for which more information was required, were read in full; (3) any studies that met relevance criteria following review of the full article proceeded to data extraction. The eligibility of each study was assessed independently by two reviewers (JS, AN; LM, ET; DH, JPM; YH, HL). Disagreements regarding eligibility were resolved by consensus.

### 2.3 Data extraction

Data from each relevant article was extracted independently by two reviewers (JS, AN; LM, ET; DH, JPM; YH, HL), using pre-tested forms. Extracted information from primary studies included: first author, publication year, title, language, study objective, host/source, location of data collection, location of lab analysis, study design, sampling method, inclusion/exclusion criteria, sample size, reported prevalence, detection method(s), tests in series/parallel, criteria for assigning case status, test characteristics, method(s) for determining test characteristics, and if *E. multilocularis* samples were sequenced and/or submitted to GenBank® (Clark et al., 2016), an open access nucleotide sequence database. When a study reported the criteria to assign case status, this was extracted and recorded. If no explicit criteria were stated, we assumed that the results of a single test were used to assign case status but classified the case definition as Not Reported when multiple tests were used. Data elements extracted from grey literature included title, first author, publication year, report type, host/source, case definition(s), reportable/notifiable directive, diagnostic method(s) used and reported prevalence.

### 2.4 Quality assessment

Only studies that evaluated diagnostic test accuracy for detection of the parasite were assessed for quality. Each study that passed through quality assessment was independently evaluated by two separate reviewers (LM, JS) using the Quality Assessment of Diagnostic Accuracy Studies 2 (QUADAS 2) tool (Whiting et al., 2011). QUADAS 2 measures risk of bias and applicability to the review question within four domains: patient selection, index test, reference standard, and flow/timing. Disagreement was settled by discussion and consensus.

### 2.5 Data synthesis

We reported the geographic location of population level *E. multilocularis* surveillance, the detection tools used by such studies and the diagnostic test characteristics of detection tools evaluated as part of Phase III or Phase III field studies. Maps display one study location per citation and were created using ArcGIS v10.6. Elements of the QUADAS 2 assessment were rated as high risk of bias, low risk of bias or unclear according to the method's recommendations for analysis (Whiting et al., 2011).

## 3 Results

### 3.1 Primary literature search

**Fig. 1:**
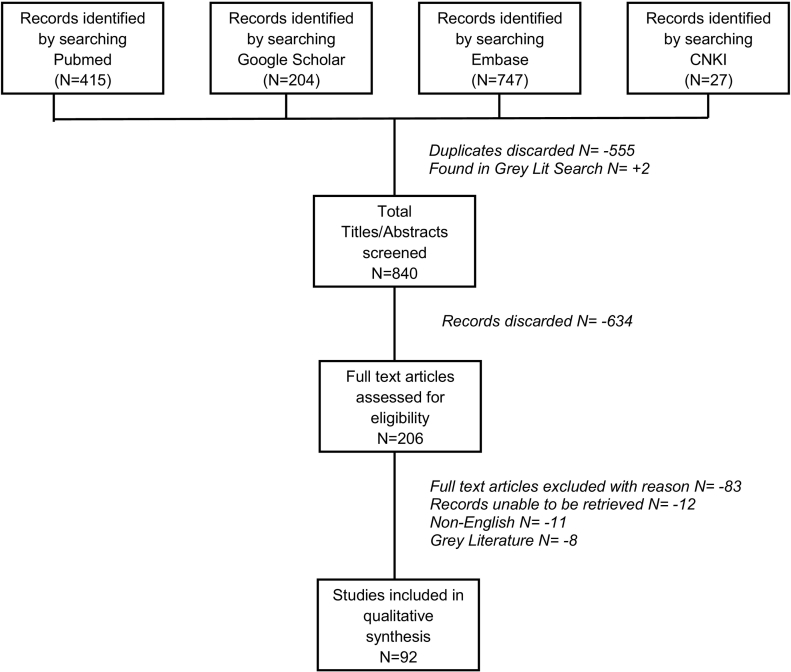
Systematic search strategy of English and Chinese language peer-reviewed literature databases (CNKI – Chinese National Knowledge Infrastructure).

**Fig. 2:**
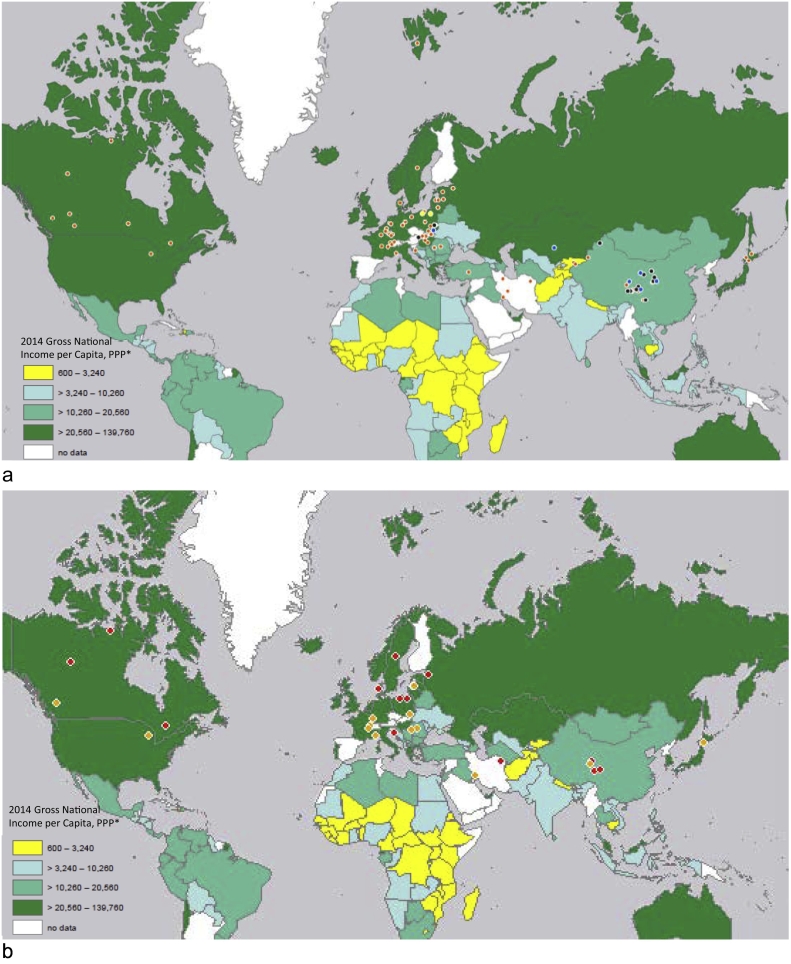
a. Economic status of countries where *Echinococcus multilocularis* surveillance of canids, humans, or the environment was reported in English or Chinese language primary literature (2008–2018). b. Economic status of countries where *Echinococcus multilocularis* surveillance included DNA sequencing and/or submission of DNA sequences into GenBank® (an open access nucleotide sequence database).

Our searches of English and Chinese language primary literature identified 1393 articles that matched our search terms for the years 2008–2018 (Fig. 1). Of these, 1295 records were excluded because they were duplicates, unavailable, did not meet inclusion criteria, or were written in a language other than English or Chinese. The primary literature search retrieved eight reports deemed to be grey literature, which were then included in grey literature relevance screening. In total, the search team extracted data from 92 peer-reviewed articles reporting *E. multilocularis* prevalence or diagnostic test evaluation in canids (N = 69; Table 2), humans (N = 14; Table 3), multiple hosts (N = 6; Table 4), or the environment (N = 3; Table 5). Prevalence estimates were most frequently reported for China (N = 21), France (N = 11), Poland (N = 10) and Canada (N = 7), with an overall dataset that spanned 27 of the 43 AE endemic countries in North America, Europe and Asia (Fig. 2a,b). The countries that reported some form of surveillance for this parasite were most frequently categorized as high (74%, 20 of 27) or upper middle (22%, 6 of 27) income by the World Bank (The World Bank, 2018). Countries not reporting data were evenly categorized as high, upper-middle, and low-middle (31%, 5 of 16, each) with one country categorized as low income. Only 34 of the 92 studies reported the location of sample analysis; of these, most research teams analyzed their samples in the same country where they were collected (85%, 29 of 34). Only two studies estimated *E. multilocularis* prevalence in a low income country (Kyrgyzstan) and these occurred through collaboration between Kyrgyz and Swiss researchers, with molecular analyses performed in Switzerland (Ziadinov et al., 2008; Ziadinov et al., 2010).

**Table 2:** Summary of data extracted from English and Chinese language primary literature reporting surveillance for *Echinococcus multilocularis* in wild and/or domestic canids (2008–2018).

Authors, year (language)	Host	Location	Study design	Sampling method	Sample size	Prevalence % (95% CI)	Detection method(s)	Case definition	Seq/submission^a^
Hanosset et al. 2008 (English)	Red foxes	Wallonoia, Southern Belgium	Cross-sectional	Convenience	990	24.55 (22.38–27.87)	(i) Intestines: mucosal scraping (ii) Cestode morphology	Morphology +ve	NA
Dyachenko et al. 2008 (English)	Dogs	Austria Denmark Germany France Italy Luxembourg Netherlands	Cross-sectional	Convenience	Austria: 812 Denmark: 517 Germany: 17894 France: 980 Italy: 249 Luxembourg: 165 Netherlands: 734	Germany: 0.24 (0.17–0.32) Other countries: 0	(i) Feces: ZnCl_2_/NaCl flotation (ii) Nested PCR (12S)	Microscopy and PCR +ve	Not sequenced
Bagrade et al. 2008 (English)	Red foxes	Latvia	Cross-sectional	Convenience	45	35.60	(i) Intestinal examination (ii) Cestode morphology (iii) PCR (*CO1, ND1, rrnS, ATP6, actII*) (iv) Sequencing	NR	Sequenced
Guislain et al. 2008 (English)	Foxes	French Ardennes region, France	Cross-sectional	Convenience	149	53 (45.4–60.6)	(i) Intestines: SCT^b^ (ii) Cestode morphology (iii) Fragment size analyses (EmsB microsatellite target)	NR	Not sequenced
Ziadinov et al. 2008 (English)	Dogs (no puppies, pregnant bitches)	At-Bashy, Naryn province, Kyrgyzstan	Cross-sectional	Cluster	466	11	(i) Arecoline purgation (ii) Cestode morphology (iii) Fecal: ZnCl_2_ sieving/flotation (v) Multiplex PCR	Morphology or PCR +ve	Not sequenced
Bourecka et al. 2008 (English)	Red foxes	Małopolskie voivodship, Poland	Cross-sectional	Convenience	214	20.1 (14.72–25.46)	(i) Intestines: IST^c^ (ii) Cestode morphology	Morphology +ve	NA
Antolova et al. 2009 (English)	Dogs (not dewormed in 4 mos)	Presov and Kosice regions, Slovakia	Cross-sectional	NR	289	2.8	(i) Feces-Sheather's sucrose flotation (ii) Copro-antigen ELISA^d^ (iii) Nested copro-PCR (12S)	PCR +ve	Not sequenced
Takumi et al. 2008 (English)	Foxes	Groningen & Limburg Provinces, The Netherlands	Cross-sectional	Convenience	1996–1997: 39 2003: 195	1996–1997: 7.7 2003: 11.7	(i) Intestines: IST (ii) PCR	NR	Not sequenced
Malczewski et al. 2008 (English)	Red foxes	Northeast & Southeast Poland	NR	NR	1514	23.8 (21.63–25.92)	(i) Intestines: IST (ii) Cestode morphology	Morphology +ve	NA
Robardet et al. 2008 (English)	Red foxes	Nancy, France	Cross-sectional	Convenience	127	30	(i) Intestines: SCT (ii) Cestode morphology	Morphology +ve	NA
Nonaka et al. 2009 (English)	Dogs	Hokkaido, Honshu, Kyushu, Japan	Cross-sectional	Convenience	Hokkaido: 4768 Honshu/Kyushu: 348	Hokkaido: 0.86 Honshu/Kyusho: 0.86	(i) Feces: Sieving/flotation (ii) Copro-antigen ELISA (iii) PCR (*CO1*, 12S, U1 snRNA)	NR	Sequenced
Bružinskaitė et al. 2009 (English)	Dogs (in AE +ve villages)	Lithuania	Cross-sectional	Convenience	240	0.8% (0.1–3)	(i) Feces: McMaster method (ii) ZnCl_2_ sieving/flotation (iii) Multiplex PCR	NR	Not sequenced
Nonaka et al. 2009 (English)	Silver foxes, red foxes, raccoon dogs, Dogs	Hokkaido, Japan	Cross-sectional	NR	Foxes: 209 Raccoon dogs: 3 Dogs: 3	Foxes: 12.9 Raccoon dogs: 0 Dogs: 0	(i) Feces: Sucrose flotation (ii) Copro-antigen ELISA (iii) PCR (*CO1*)	PCR +ve	Not sequenced
Hurnikova et al. 2009 (English)	Red foxes, Raccoon dogs	Tatra National Park, Slovakia	Cross-sectional	Convenience	Red foxes: 328 Raccoon dogs: 2	Red foxes: 42.7 Raccoon dogs: 50	(i) Intestines: SCT (ii) Cestode morphology	Morphology +ve	NA
Bagrade et al. 2009 (English)	Wolves	Latvia	NR	NR	34	5.9	(i) Intestinal examination (ii) Cestode morphology	Morphology +ve	NA
Barabasi et al. 2010 (English)	Red foxes	Romania	Cross-sectional	Convenience	561	4.8 (3.2–6.9)	(i) Intestines: Sedimentation & mucosal scraping (ii) Cestode morphology (iii) Multiplex PCR (iv) Sequencing	NR	Sequenced
Casulli et al. 2010 (English)	Red foxes	Hungary	Cross-sectional	NR	840	10.7 (9.7–11.7)	(i) Intestines: SCT (ii) Cestode morphology (iii) Fluorescent PCR & fragment size analyses (EmsB microsatellite target)	NR	Not sequenced
Stien et al. 2010 (English)	Arctic foxes	Svalbard Islands, Norway	Cross-sectional	Convenience	353	8.5 (6–11.9)	(i) Intestines: IST (ii) Cestode morphology	Morphology +ve	NA
Wang et al. 2010 (English)	Dogs	Shiqu County, Ganzi Tibetan Autonomous Prefecture, China	NR	Clustered, non-random	228	14.80	(i) Arecoline purgation (ii) Cestode morphology (iii) Copro-PCR (12S)	Copro-PCR +ve	Not sequenced
Ziadinov et al. 2010 (English)	Red foxes	Naryn Oblast, Kyrgyzstan	Cross-sectional	Convenience	151	63.6 (55.4–71.3)	(i) Intestines – SCT (ii) Cestode morphology	Morphology +ve	NA
Siko et al. 2011 (English)	Red foxes	Romania	Cross-sectional	Convenience	561	4.8 (3.2–6.9)	(i) Intestines: SCT and/or mucosal scraping (ii) Cestode morphology (iii) Multiplex PCR (iv) Sequencing	NR	Sequenced
Beiromvand et al. 2011 (English)	Dogs, foxes, wolves	Chenaran,Razavi Khorasan Province, Iran	Cross-sectional	Convenience	Dogs: 77 Foxes: 3 Wolf: 1	Dogs: 6.5 (2.8–14.3) Foxes & wolf: 100 (78.5–100)	Dogs: (i) Fecal ZnCl_2_ sieving/flotation (ii) Multiplex PCR Foxes & wolf: (i) Intestines: IST or SCT (iii) Cestode morphology	NR	Sequenced and submitted
Karamon et al. 2011 (English)	Red foxes	Świętokrzyskie & Lubelskie Provinces, Poland	Cross-sectional	NR	353	13.6	Intestines: SCT	SCT +ve	NA
Umhang et al. 2011 (English)	Foxes	France	Cross-sectional	NR	358	32.7	Reference: SCT Index: SSCT^e^	SCT +ve	NA
Miterpakova et al. 2011 (English)	Red foxes	Slovakia	Cross-sectional	Stratified cluster	4761	30.3	Intestines: SCT	SCT +ve	NA
Umhang et al. 2012 (English)	Dogs	Meuse and Haute-Saone, France	Cross-sectional	Convenience	Meuse: 493 Haute-saone: 367	Meuse: <1 Haute-saone: <0.75	(i) Fecal ZnCl_2_ sieving/flotation (ii) PCR (ND1)	PCR +ve	Not sequenced
Catalano et al. 2012 (English)	Coyotes	Alberta, Canada	Cross-sectional	Convenience	91	25.30	(i) Intestinal sieving (ii) Cestode morphology (iii) Multiplex PCR	Morphology and PCR +ve	Not sequenced
Jiang et al. 2012a (English)	Tibetan foxes, Red foxes	Shiqu County, China	Cross-sectional	Convenience	184	35	Reference: RFLP-PCR Index: (i) Nested multiplex copro-PCR (*CO1*) (ii) Sequencing	NR	Sequenced and submitted
Compte et al. 2012 (English)	Foxes	France	Cross-sectional	Stratified	3307	17 (16–19)	Intestines: SSCT	SSCT +ve	NA
Jiang et al. 2012b (Chinese)	Tibetan foxes	Yunbo Gou, Shiqu County, Sichuan Province, China	Cross-sectional	Cluster	120	19	Nested duplex copro-PCR	PCR +ve	Sequenced and submitted
Takahashi et al. 2013 (English)	Foxes	Nemuro peninsula, Hokkaido, Japan	Cross-sectional	Simple random	Bait zone: 411 Control zone: 163	Pre-bait^f^ (1994–1999): Bait: 49.4 (43.7–55) Control: 70.5 (60.2–79.2) Post-bait: (2003–2006): Bait: 15.8 (7.9–28.4) Control: 65 (40.9–83.7)	(i) Intestines: mucosal scraping (ii) Cestode morphology	Morphology +ve	NA
Tolnai et al. 2013 (English)	Red foxes	Hungary	Cross-sectional	Random	2008: 840 2012: 772	2008: 10.7 (9.7–11.7) 2012: 7.9 (6.9–8.9)	(i) Intestines: SCT (ii) Cestode morphology (iii) Microsatellite analysis (iv) PCR (12S)	Morphology +ve	Not sequenced
Gesy et al. 2013 (English)	Red foxes, coyotes	Quesnel, Canada	Cross-sectional	Convenience	Red foxes: 6 Coyotes: 27	Coyotes: 71 Foxes: 17	(i) Intestines: SFCT^g^ (ii) Cestode morphology (iii) PCR (*ND1, ND2, COB, CO1*) (iv) Sequencing	Morphology and PCR +ve	Sequenced
Mobedi et al. 2013 (English)	Dogs, red foxes	Moghan Plain, Iran	Cross-sectional	NR	Dogs: 59 Red foxes: 84	Dogs: 0 Red foxes: 0	(i) Copro-antigen ELISA (ii) Nested copro-PCR (12S)	NR	Not sequenced
Moss et al. 2013 (English)	Dogs	Shiqu and Yajiang counties, China	Cohort	NR	592	11.20	(i) Copro-antigen ELISA (ii) Copro-PCR (*ND1*)	NR	Not sequenced
Li et al. 2013 (English)	Tibetan sand foxes and red foxes	Qinghai, China	Cross-sectional	Convenience	Intestines: 36 Feces: 70	Intestines: 3 Feces: 1.4	(i) Intestinal examination (ii) Feces-Sucrose flotation (iii) PCR (*CO1*) (iv) Sequencing	NR	Sequenced
Comte et al. 2013 (English)	Foxes	Annemasse & Pontarlier, France	Cross-sectional	Purposive	Annemasse: 700 Pontarlier: 700	Pre-bait^f^ (2006) Annemasse: 13.3 Pontarlier: 10.9 Post-bait (2007–2009) Annemasse: 2.2 Pontarlier: 7.1	Copro-antigen ELISA	ELISA +ve	NA
Schurer et al. 2014 (English)	Wolves	Saskatchewan (SK), Manitoba (MB), Northwest Territories (NT)	Cross-sectional	Convenience	SK: 17 MB: 3 NT: 73	SK: 23.5 MB: 67 NT: 8.2	(i) Intestines: SFCT (ii) Cestode morphology (iii) PCR (*CO1, ND1*) (iv) Sequencing	PCR +ve	Sequenced and submitted
Umhang et al. 2014 (English)	Dogs	Annemasse and Pontarlier, France	Cross-sectional	NR	817	0.5 (0.1–1.3)	(i) Fecal sieving/flotation (ii) Multiplex PCR (iii) Sequencing	PCR +ve	Sequenced
Isaksson et al. 2014 (English)	Red foxes	Eastern Switzerland and Sweden	Cross-sectional	NR	Switzerland: 177 Sweden: 2158	NA	Reference: Intestines: SCT Index: Fecal Magnetic Capture RT-PCR	SCT +ve	Not sequenced
Denzin et al. 2014 (English)	Red foxes	Saxony-Anhalt, Germany	Cross-sectional	NR	1998–2005: 1882 2006–2010: 2307	1998–2005: 13.6 (11.6–15.6) 2006–2010: 23.4 (21.2–25.7)	(i) Intestinal Smear Technique (ii) Cestode morphology	‘Smear’ +ve	NA
Maas et al. 2014 (English)	Red foxes, Dogs (>6 mos, not dewormed in 1 mo)	South Limburg, Maastricht, The Netherlands	Cross-sectional	Convenience	Red foxes: 37 Dogs: 142	Red foxes: 59 (43–74) Dogs: 0	Red foxes: (i) IST (ii) Nested PCR Dogs: Copro-qPCR	Red foxes: IST or PCR +ve Dogs: qPCR +ve	Not sequenced
Liccioli et al. 2014 (English)	Coyotes	Calgary, Canada	Cross-sectional	Convenience	385	21.42	(i) Fecal ZnCl_2_ sieving/flotation (ii) PCR	PCR +ve	Not sequenced
Karamon et al. 2014 (English)	Red foxes	Poland	Cross-sectional	Convenience	1546	16.5 (14.7–18.4)	Intestines: SCT	SCT +ve	NA
Gesy et al. 2014 (English)	Arctic foxes Red foxes Coyotes	Canada	Cross-sectional	Convenience	Arctic foxes: 404 Red foxes: 6 Coyotes: 48	Arctic foxes: 0.74 Red foxes: 17 Coyotes: 58	(i) Fecal Sucrose Flotation (ii) Intestines: SFCT (iii) Simplex/multiplex PCR (iv) Sequencing	NR	Sequenced and submitted
Ma et al. 2014 (Chinese)	Red foxes	Zhaosu basin, China	Cross-sectional	Stratified cluster	6	50	(i) Intestinal examination (ii) Cestode morphology	Morphology +ve	Not sequenced
Karamon et al. 2015 (English)	Red foxes	Śląskie, Opalskie, Lubelskie, Podkarpackie, Poland	Cross-sectional	Convenience	500	Śląskie: 11.7 (6.7–19.4) Opalskie: 3.9 (1.6–8.4) Podkarpackie: 54.6 (46.7–62.3) Lubelskie: 18.9 (12.0–28.3) Overall: 23.6	Intestines: SCT	SCT +ve	NA
Melotti et al. 2015 (English)	Coyotes, red foxes, grey foxes	Michigan, USA	Cross-sectional	Convenience	Coyotes: 223 Grey foxes: 45 Red foxes: 34	Coyotes: 0.4 Red foxes: 0 Grey foxes: 0	All canids: (i) Intestines: SCT (ii) Multiplex PCR (iii) Sequencing	NR	Sequenced
Villeneuve et al. 2015 (English)	Shelter dogs (not dewormed in 5 mos)	Canada	Cross-sectional	Quota	1086	0	(i) Fecal sucrose flotation (ii) Multiplex PCR	PCR +ve	Not sequenced
Laurimaa et al. 2015 (English)	Raccoon dogs	Estonia	Cross-sectional	Convenience	249	1.6 (0.4–4.1)	(i) Intestines: SCT (ii) Cestode morphology (iii) PCR (*Ile/Lys*) (iv) Sequencing	NR	Sequenced and submitted
Laurimaa et al. 2016 (English)	Red foxes	Estonia	Cross-sectional	Convenience	111	31.5 (22.7–40.3)	(i) Intestines: SCT (ii) Cestode morphology	Morphology +ve	NA
Bagrade et al. 2016 (English)	Red foxes, racoon dogs	Latvia	Cross-sectional	NR	Red foxes: 538 Racoon dogs: 407	Red foxes: 17.1 (13.9–20.3) Racoon dogs: 8.1 (5.5–10.8)	All canids: (i) Intestines: SCT (ii) Cestode morphology (iii) Multiplex PCR (iv) PCR (*CO1*) (v) Sequencing	NR	Sequenced
Miller et al. 2016 (English)	Red fox	Katrineholm, Uddevalla, Gnesta/Nyköping, Vetlanda/Växjö, Sweden	Cross-sectional	Purposive	714	5.7 (4.2–7.7)	(i) Fecal sieving/flotation (ii) Multiplex PCR (iii) Sequencing	PCR and sequence +ve	Sequenced and submitted
Umhang et al. 2016 (English)	Wild: foxes Captive: red foxes, Alaskan tundra wolves, Eurasian wolves	Moselle, France	Wild: cross-sectional Captive: cohort	Purposive	Captive: Foxes:3 Alaskan wolves: 35 Eurasian wolves: 75 Wild foxes: Feces (in/out park): 142 Intestines: 5	Captive: Eurasian wolves: 1.3 Red fox: 0 Alaskan wolves: 0 Wild foxes: Outside park: 20.6 (13.1–30) Inside park: 17.8 (8–32.1) Intestines: 60	Feces: (i) Sieving/flotation (ii) Multiplex PCR (iii) Sequencing Intestines: (i) SSCT (ii) EmsB microsatellite analysis	NR	Sequenced
Comte et al., 2017 (English)	Foxes	Nancy, France	Cross-sectional	Systematic	445	57%	Intestines: SSCT	SSCT +ve	NA
Frey et al. 2017 (English)	Dogs (clinical AE cases, presumed uninfected, negative controls)	Switzerland	Diagnostic investigation	Presumed uninfected - random; Clinical AE and negative controls - NR	75	NA	Reference: Abdominal ultrasound Index (Serology): (i) ELISA (EmVF, Em2-antigen, rEm18, rEm95) (ii) In-house Western Blot (iii) Anti-Echinococcus EUROLINE-Western Blot (IgG, rEm18, rEm95, rEgAgB)	Ultrasound +ve	NA
Schuster & Shimalov 2017 (English)	Raccoon dogs, red foxes	Uckermark district, Brandenburg state, Germany	Cross-sectional	Convenience	Raccoon dogs: 101 Red foxes: 144	Raccoon dogs: 0.99 Red Foxes: 1.39	(i) Intestines: Sedimentation Technique (ii) Cestode morphology	Morphology +ve	NA
Maksimov et al. 2017 (English)	Foxes	NR	NR	NR	120	NA	Reference: intestines: IST Index 1: (i) ZR Faecal DNA MiniPrep™ (ii) FastDNA® SPIN Kit for Soil (iii) QIAamp® Fast DNA Stool MiniKit (iv) NucleoSpin®Soil Index 2: (i) PCR (*rrn*S), (ii) iQS-qPCR (*rrn*L) (iii) QT-qPCR (*rrn*L)	NR	Not sequenced
Poulle et al. 2017 (English)	Dogs, red foxes	Ardennes, France	Cross-sectional	Convenience	Dogs: 18 Red foxes: 69	Dogs: 11.1 (1.4–34.7) Red foxes: 34.8 (23.5–47.6)	Copro-qPCR (*rrn*L)	qPCR +ve	Not sequenced
Kohansal et al. 2017 (English)	Stray dogs	Zanjan Province, Iran	Cross-sectional	NR	450	0	(i) Fecal Formalin ethyl acetate concentration test (ii) ZnCl_2_ sieving/flotation (iii) Multiplex PCR	PCR +ve	Not sequenced
Hermosilla et al. 2017 (English)	Wolves	Gorski Kotar region, Croatia	Cross-sectional	NR	400	0	(i) Fecal Sodium acetate formalin test (ii) Nested PCR (12S) (iii) Sequencing	NR	Sequenced and submitted
Otero-Abad et al. 2017 (English)	Red foxes	Switzerland	Cross-sectional	Convenience	300	59.5 (43.1–66.4)	Reference: Intestines – SCT Index (i): Polyclonal copro-antigen ELISA Index (ii): Monoclonal copro-antigen ELISA Index (iii): Multiplex PCR	SCT +ve	Not sequenced
Schurer et al. 2018 (English)	Wolves, coyotes, red foxes, Arctic foxes	Quebec Canada (QC), Maine USA (ME)	Cross-sectional	Convenience	QC: 284 ME: 23	QC: 0 ME: 0	All canids: (i) Intestines: SFCT (ii) Simplex/multiplex PCR (*CO1, ND1*) (iii) Sequencing	NR	Sequenced and submitted
Petersen et al. 2018 (English)	Red foxes, Raccoon dogs	Denmark	Cross-sectional	Convenience	Red foxes: 1073 Raccoon dogs: 272	Red foxes: 1.8 Raccoon dogs: 0.7	Intestines: (i) SCT (ii) Cestode morphology Feces: Magnetic Capture RT- PCR	Morphology or PCR + ve	Sequenced and submitted
Massolo et al. 2018 (English)	Wolves, dogs	Parco Regionale delle Alpi Liguri, Italy	Cross-sectional	Convenience	Dogs: 32 Wolves: 120	Dogs: 12.5 Wolves: 4.2	All canids: (i) Feces - ZnCl_2_ sieving/flotation (ii) PCR (*ND1, COB*) (iii) Sequencing	PCR +ve	Sequenced
Gurler et al. 2018 (English)	Red foxes	Central Anatolia and Thrace, Turkey	Cross-sectional	Random	405	1.90	(i) Fecal flotation (ii) Multiplex PCR	PCR +ve	Not sequenced
Beiromvand et al. 2018 (English)	Domestic dogs	Ahvaz County, Khuzestan Province, Iran	Cross-sectional	Simple random	167	0	(i) ZnCl_2_ sieving/flotation (ii) Multiplex PCR	NR	Sequenced
Knapp et al. 2018 (English)	Red foxes, Dogs	Franche-Comté and Ile-de-France, France	Cross-sectional	Systematic	Red foxes: 590 Dogs: 1858	Red foxes: 27.9 (23.8–32.4) Dogs: 1.1 (0.4–3.0)	(i) Copro-qPCR (*rrn*L) (ii) Sequencing	Sequence +ve	Sequenced
Liu et al. 2018 (English)	Dog	Xiji County, Ningxia Hui Autonomous Region, China	Cross-sectional	Convenience	750	14.1	(i) Multiplex copro-PCR (*ND5*)	Copro-PCR +ve	Not sequenced

NR - not reported; NA - not applicable.

a *E. multilocularis* PCR products sequenced and submitted to GenBank.

b SCT - Sedimentation and Counting Technique.

c IST - Intestinal Scraping Technique.

d Enzyme-linked Immunosorbent Assay.

e SSCT - Segmental Sedimentation and Counting Technique.

f Praziquantel-based baits.

g SFCT - Sedimentation, Filtration, and Counting Technique.

**Table 3:** Summary of data extracted from English and Chinese language primary literature reporting surveillance for *Echinococcus multilocularis* in humans (2008–2018).

Authors, year (language)	Host	Location	Study design	Sampling method	Sample size	Prevalence % (95%CI)	Detection method(s)	Case definition	Seq/submission^a^
Yang et al. 2008 (English)	Children (7–18 yrs)	Xiji County, China	Cross-sectional	Purposive	861	US^b^: 0 Serology: 18	(i) US (ii) Serology - EmP-ELISA^c^	ELISA +ve	NA
Zhao et al. 2008 (Chinese)	Humans	Gannan Tibetan Autonomous Prefecture, Gansu Province, China	Cross-sectional	Stratified	1040	0.29	(i) US (ii) Serology - ELISA	US and ELISA +ve	NA
Han et al. 2009 (Chinese)	Humans	Darlag County of Qinghai Province, China	Cross-sectional	Stratified	1723	8.20	(i) US (ii) Serology – IHA^d^	US or IHA +ve	NA
Wang et al. 2009 (Chinese)	Humans	Hobukesar Mongolian Autonomous County, Xinjiang, China	Cross-sectional	Cluster	712	0.30	(i) US (ii) Serology	US or ELISA +ve	NA
Feng et al. 2010 (English)	Humans	Xiji County in Ningxia Hui Autonomous Region, Ganzi County in Sichuan Province, and Dingqing County in Tibet Autonomous Region, China	NR	NR	3191	3.38	(i) Reference: US (ii) Index: Em2-DIGFA (Dot immunogold filtration assay)	US +ve	NA
Li et al. 2010 (English)	Humans	Sichuan Province, China	Cross-sectional	Convenience	10,186	3.05	(i) US (i) Serology- Em18-ELISA	US +ve	NA
Poeppel et al., 2013 (English)	Humans (18–60 yrs)	Austria	Cross-sectional	Convenience	1046	0	(i) Serology -Em-ELISA (ii) Serology - Western Blot	ELISA and Western Blot +ve	NA
Liu et al. 2014 (Chinese)	Children (4–18 yrs)	Xiji county, Ningxia Hui Autonomous Region, China	Cross-sectional	Stratified cluster	1772	6.72	Serology - ELISA	ELISA +ve	NA
Cisak et al. 2015 (English)	Humans (rural east Poland)	Bialystok, Lublin and Rzeszow, Poland	Cross-sectional	Purposive	172	1.7	(i) Serology- Em2^Plus^ ELISA (ii) Serology - Western Blot	ELISA and Western Blot +ve	NA
Cai et al. 2017 (English)	Students (6–16 yrs)	Golog Tibetan Autonomous Prefecture, China	Cross-sectional	NR	11,260	1.29	(i) US (ii) Serology (IgG)	US and serology +ve	NA
Han et al. 2017 (Chinese)	Children (6–12 yrs)	Yushu and Guoluo prefectures, Qinghai Province, China	Cross-sectional	Cluster	US: 19629 Serology: 9888	US: 1.13 Serology: 12.59	(i) US (ii) Serology	US or serology +ve	NA
Cadavid Restrepo et al. 2018 (English)	Children (6–18 yrs)	Xiji County, Ningxia Hui Autonomous Region, China	Cross-sectional	Cluster	5110	12.20	(i) US (ii) Serology: EmP-ELISA	ELISA +ve	NA
Han et al. 2018 (English)	Students (6–18 yrs)	Yushu and Guoluo Tibetan Autonomous Prefectures, China	Cross-sectional	Multistage cluster	US: 19629 Serology: 16969	1.10	(i) US (ii) Serology (IgG-ELISA)	US +ve	NA
Gao et al. 2018 (English)	Humans	Ganzi Tibetan Autonomous Prefecture	Cross-sectional	Convenience	1502	13.45	(i) Reference: US (ii) Index: Serology - Antibody Gold Immuno-chromatographic assay	US +ve	NA

NR - not reported; NA - not applicable.

a *E. multilocularis* PCR products sequenced and submitted to GenBank.

b Abdominal ultrasound.

c Enzyme-linked Immunosorbent Assay.

d Indirect Hemagglutination Test.

**Table 4:** Summary of data extracted from English and Chinese primary literature reporting surveillance for *Echinococcus multilocularis* in canids and humans (2008–2018).

Authors, year (language)	Host	Study location	Study design	Sampling method	Sample size	Prevalence % (95% CI)	Detection method(s)	Case definition	Seq/submission^a^
Torgerson et al. 2009 (English)	Humans Dogs	Jalanash, Kazakhstan	Cross-sectional	Convenience	Humans: 3126 Dogs: 632	Humans: 0 Dogs: 5	Humans: (i) Abdominal ultrasound (ii) Serology: Em2G11-ELISA^b^ Dogs: Arecoline purgation	NR	NA
Han et al. 2015 (English)	Humans Dogs	Minle County, China	Cross-sectional	Convenience	Humans: 362 Dogs: 356	Humans: 0.29 Dogs: 0	Humans: (i) Abdominal ultrasound (ii) Serology: Colloidal gold rapid diagnostic kit Dog feces: Kato-Katz technique	NR	NA
Ma et al. 2015 (English)	Humans (echinococcosis surgical cases) Dogs, Tibetan foxes	Qinghai, China	Cross-sectional	NR	Humans: 163 Dogs: 21 Foxes: 2	Humans: 10.4 Dogs: 76 Foxes: 0	Humans and canids: (i) Simplex PCR (*CO1*) (ii) Sequencing	NR	Sequenced and submitted
Xu et al. 2015 (Chinese)	Children (4–18 years) Dogs	Haiyuan Counties and Guyuan District, Ningxia Hui Autonomous Region, China	Cross-sectional	Stratified random	Humans: 5706 Dogs: 2175	Humans: Xiji: 8.38 Haiyun: 7.03 Guyuan: 20.48 Dogs: Xiji: 6.40 Haiyun: 1.52 Guyuan: 3.37	Humans: Serology-:Em-ELISA Dogs: Copro-PCR	Humans: ELISA +ve Dogs: PCR +ve	Not sequenced
Karamon et al. 2016 (English)	Humans (*E. multilocularis* +ve dog owners) Dogs, red foxes	Podkarpackie Province, Poland	Cross-sectional	Purposive	Humans: 8 Dogs: 148 Foxes: 59	Humans: 0 Dogs: 1.4 (0.4–4.8) Foxes: 46	Humans: Serology - IgG-ELISA Dogs: Copro-PCR (12S) Foxes: Intestines – SCT^c^	NR	Sequenced
Sen-Hai et al. 2008 (English)	Humans (≥5 years) Dogs	Jiuzhi County, China	Cross-sectional	Convenience	Humans: Ultrasound: 1549 Serology: 1113 Stray dogs: 12 Dog feces: 149	Humans: 2.5 Stray dogs: 8 Dog feces: 0	Humans: (i) Abdominal ultrasound (ii) Serology –IHA^d^ (iii) Immunoblot Dogs: (i) Necropsy (ii) Copro-antigen ELISA (iii) Wisconsin flotation (iv) Copro-PCR (12S) (v) RFLP^e^ (*Ase*I, *Ssp*I)	NR	Not sequenced

NR - not reported; NA - not applicable.

a *E. multilocularis* PCR products sequenced and submitted to GenBank.

b Enzyme-linked Immunosorbent Assay.

c SCT - Sedimentation and Counting Technique.

d IHA - Indirect Hemagglutination Assay.

e Restriction Fragment Length Polymorphism.

**Table 5:** Summary of data extracted from primary literature surveillance for *Echinococcus multilocularis* on environmental samples (2008–2018).

Authors, year (language)	Host/source	Study location	Study design	Sampling method	Sample size	Prevalence %	Detection method(s)	Case definition	Seq/submission^a^
Szostakowska et al. 2014 (English)	Soil from kitchen gardens, farmyards, arable fields, forests, and areas near fox dens/lairs	Varmia-Masuria Province, Poland	Cross-sectional	Purposive	62	11.3	(i) ZnCl_2_ flotation (ii) Nested PCR (12S) (iii) Sequencing	PCR +ve	Sequenced and submitted
Lass et al. 2015 (English)	Fruits, vegetables, mushrooms from forests, kitchen gardens and plantations	Varmia-Masuria Province, Poland	Cross-sectional	Convenience	103	23.3	(i) ZnCl_2_ flotation/sieving (ii) Nested PCR (12S) (iii) Sequencing	PCR +ve	Sequenced and submitted
Lass et al. 2017 (English)	Fruits, vegetables, mushrooms from forests, kitchen gardens and plantations	Pomerania Province, Poland	Cross-sectional	Convenience	104	6.73	(i) ZnCl_2_ flotation/sieving (ii) Nested PCR (12S) (iii) Sequencing	PCR +ve	Sequenced and submitted

NR - not reported.

a *E. multilocularis* PCR products sequenced and submitted to GenBank.

### 3.2 Surveillance for *E. multilocularis* in canids

Our search collected 75 primary research studies that carried out case detection and population surveillance of *E. multilocularis* cestodes in wild and domestic canids (Table 2). Of these, six studies reported simultaneous surveillance in canids and humans (Table 4). Canid surveillance was most frequently reported from France and China (N = 11 each), followed by Canada (N = 7), Poland (N = 6), and Iran (N = 5) (Fig. 2a). Many studies reported prevalence estimates for multiple canid species, and out of all canid or canid/human studies the majority focused on red foxes (49%), followed by dogs (32.5%) and foxes (unspecified, 12.5%). Just under 10% reported on each of coyotes, raccoon dogs and wolves, and our search also found studies that assessed prevalence in silver foxes, arctic foxes, grey foxes, Tibetan foxes, and a single study that reported on captive canids. Most studies used a variety of diagnostic techniques to identify and confirm *E. multilocularis* infection, including necropsy or arecoline hydrobromide purgation followed by morphological identification of cestodes, copro-PCR, fecal sieving/flotation followed by PCR, and coproantigen ELISA (Enzyme-Linked Immunosorbent Assay). Post-mortem examination of canid intestinal tracts was employed using various techniques (Sedimentation and Counting Technique - SCT, Intestinal Scraping Technique - IST, Segmental Sedimentation and Counting Technique - SSCT, Sedimentation, Filtration and Counting Technique -SFCT) to collect *E. multilocularis* adult cestodes for species level identification (N = 44). Molecular identification of taeniid eggs or *Echinococcus* cestodes, either as a stand-alone assay or to confirm morphological results, was conducted by 55 research teams. These methods included a variety of PCR techniques - conventional simplex, nested, multiplex, copro-, fluorescent, qPCR, RFLP-PCR, magnetic capture RT-PCR, as well as microsatellite analysis. Various loci were targeted, including cytochrome *c* oxidase subunit 1 (*co1*), NADH dehydrogenase subunits 1 and 5 (*nd1, nd5*), ATPase subunit 6 (*atp6*), cytochrome *b* (*cob*), and the small and large subunit rRNA genes (*rrn*S, *rrn*L). Multiplex PCR was the single most popular molecular technique (N = 20), with the majority carried out using Cest1/Cest2 primers (*nd1*) to differentiate *E. multilocularis* from *E. granulosus* and *Taenia* spp. (Trachsel et al., 2007). Only two studies used alternative multiplex primers (Jiang et al., 2012; Liu et al., 2018). Of the studies using PCR to detect *E. multilocularis* in canids, approximately half (57%, 25 of 44) sequenced PCR products and one-third (34%, 15 of 44) submitted these nucleotide sequences into GenBank®. The highest number of records that sequenced and/or submitted *E. multilocularis* sequences originated from Canada (Gesy et al., 2013, Gesy et al., 2014; Schurer et al., 2014; Schurer et al., 2018), China (Jiang et al., 2012; Ma et al., 2015; Li et al., 2013; 蒋韦斌, 2012), and Poland (Karamon et al., 2016; Lass et al., 2015; Lass et al., 2017; Szostakowska et al., 2014) (N = 4 each), followed by France (Umhang et al., 2016; Knapp et al., 2018; Umhang et al., 2014) (N = 3). In addition to these morphological and molecular techniques, our review identified eight studies from six countries where immunological tests (copro-antigen ELISA) were used to screen fecal samples collected from wild foxes (N = 4) and/or domestic dogs (N = 6) (Sen-Hai et al., 2008; Otero-Abad et al., 2017; Nonaka et al., 2009a, Nonaka et al., 2009b; Mobedi et al., 2013; Comte et al., 2013; Antolova et al., 2009; Moss et al., 2013) for *E. multilocularis*. Although canid studies often did not explicitly state their sample collection strategy, most appeared to use convenience sampling and a cross-sectional approach. Furthermore, 36% (29/80) of studies reporting surveillance of *E. multilocularis* in canids did not describe the case definition (Table 2, Table 3).

### 3.3 Surveillance for *E. multilocularis* in humans

In total, 20 studies from four countries (China, Kazakhstan, Poland, Austria) conducted population surveillance for AE in people (Table 3, Table 4). Prevalence based on abdominal ultrasound and serology was estimated to be 0–13.45% in China (Yang et al., 2008; Gao et al., 2018), and 0% in Kazakhstan (Torgerson et al., 2009). Seroprevalence was estimated to be 0% in Austria (Poeppl et al., 2013) and 0–1.7% in Poland (Karamon et al., 2016; Cisak et al., 2015). Most case definitions (70%, 14/20) were based on abdominal ultrasound and confirmatory serology (ELISA and/or Western Blot), while the remainder were based on serology only. Case definitions for AE were not reported for 30% of studies where humans were surveyed for infection (Ma et al., 2015; Karamon et al., 2016; Sen-Hai et al., 2008; Torgerson et al., 2009; Han et al., 2015; 许阳阳 et al., 2015). Case definitions were reported or implied for all studies of human patients (Table 3) but were only provided for one study that addressed human/canid infection (Table 4). A variety of sampling methods were reported including convenience, purposive, randomized, and cluster. Only one study sequenced AE cyst tissue removed from human patients and submitted the DNA sequences to GenBank® (Ma et al., 2015).

### 3.4 Surveillance for *E. multilocularis* in the environment

Two studies conducted surveillance for *E. multilocularis* eggs in food (Lass et al., 2015; Lass et al., 2017), and one conducted surveillance in soil between 2008 and 2018 (Szostakowska et al., 2014; Table 5). All three studies collected taeniid eggs using ZnCl_2_ flotation, identified the eggs using nested PCR and sequencing, and submitted amplified PCR product sequences into Genbank®. The reported contamination levels ranged from 6.73% to 23.3% in Poland where the studies took place; however, these levels have been called into question.

### 3.5 *E. multilocularis* diagnostic test evaluations

Our review identified nine studies that conducted Phase III or Phase III field surveillance to evaluate the diagnostic accuracy of techniques to detect *E. multilocularis* in people, canids or the environment (Table 6). Seven of these protocols screened canids for cestode or metacestode infection, and were categorized as assays for (i) intestinal examination (N = 1), (ii) fecal analysis (N = 5), or (iii) serology (N = 1):

(i) The SSCT is a modification of the gold standard SCT protocol that reduces processing time by examining cestode predilection sites in the intestinal tract (Umhang et al., 2011). The SSCT has a high sensitivity (<98.3%) and specificity compared to SCT (both depend on the intestinal segment), but lower accuracy in estimating infection intensity.
(ii) Fecal analysis is a non-invasive method of sampling canids; however, the eggs shed by *E. multilocularis* cestodes are morphologically indistinguishable for those of other taeniid species and molecular analyses can lack sensitivity due to inconsistent shedding of eggs and the difficulty of extracting DNA from taeniid eggs. We identified one study that aimed to optimize molecular detection by testing different combinations of commercially available kits for DNA extraction with PCR and qPCR protocols (Maksimov et al., 2017). Using IST as the reference standard, the authors identified QIAamp® Fast DNA Stool Mini Kit with qPCR (using QuantiTect® Multiplex-Master Mix) as having the highest sensitivity (97%) of the combinations tested. An alternative technique, DNA fishing using magnetic probes to extract DNA in combination with RT-PCR, is less sensitive (88.2%) relative to the SCT but is appealing for mass surveillance as it can be partially automated for high throughput (Isaksson et al., 2014). This systematic review identified other techniques, such as arecoline purgation, copro-antigen ELISAs and PCR, which had even lower sensitivity but that generally had acceptable specificity (close to 100%; Maksimov et al., 2017; Ziadinov et al., 2008; Otero-Abad et al., 2017).
(iii) Lastly, an evaluation of native and recombinant antigens highlighted EmVF-Western Blot and rEm95-ELISA as two highly sensitive (100%) and specific (100% and 98%, respectively) options for serological diagnosis of AE in canids (Frey et al., 2017).

**Table 6:** Diagnostic test characteristics and resource requirements of protocols identified in this systematic review for surveillance of *Echinococcus multilocularis* in canids, humans, and/or the environment (2008–2018).

Diagnostic test	Through-put^a^	Equipment requirements	Sn% (95%CI)^b^	Sp% (95%CI)^c^	Notes	Citation
Canids - post-mortem intestinal examination						
Sedimentation & Counting Technique (SCT)	Low	Biosafety space and/or −80 °C freezer, microscope	88.5 (82.7–93.4)	100	-Requires skilled microscopist -Sample quality impacts cestode identification -Quantifying worm burden can be time intensive	Otero-Abad et al., 2017
Segmental Sedimentation & Counting Technique	Low	Biosafety space and/or −80 °C freezer, microscope	56.4–98.3 (depending on segment)	<100	-See SCT notes -Selective examination of *E. multilocularis* predilection sites to increase throughput -Lower intensity estimate accuracy than SCT	Umhang et al., 2011

Canids – fecal examination						
Arecoline hydrobromide purgation	Low	Microscope	21 (11–34)	100	-Not all canids purge after arecoline dose -Canids must be restrained -Adverse effects possible; contraindicated for pregnant bitches, puppies, and older canids	Ziadinov et al., 2008
Taeniid egg recovery/multiplex PCR^d^	Low	−80 °C freezer, centrifuge, PCR thermocycler, gel electrophoresis system, UV visualization	(1) 50 (29–72) (2) 54.8 (48.5–61)	(1) 100 (97–100) (2) 93.4 (87.3–99.1)	-Requires skilled technician -Detection depends on egg recovery assay, DNA extraction technique, and PCR protocol	(1) Ziadinov et al., 2008; (2) Otero-Abad et al., 2017
Copro-DNA/PCR (various DNA extraction kits/PCR system combinations)	Low	−80 °C freezer, microcentrifuge, PCR/qPCR system	QIAamp/QT-qPCR: 81 QIAamp/PCR: 52	QIAamp/QT-qPCR: 100 QIAamp/PCR: 100	-Requires skilled technician -DNA extraction kits limited to 0.15–0.5 g/reaction -Copro-inhibitors impact extraction	Maksimov et al., 2017
Copro-antigen ELISA^e^	High	Incubator, ELISA plate washer, ELISA reader	Monoclonal: 63.2 (55.3–70.8) Polyclonal: 56 (48.0–63.9)	Monoclonal: 70.0 (60.1–79.4) Polyclonal: 65.9 (55.8–75.6)	-Requires skilled technician -Detects patent and pre-patent infections -Sensitivity depends on worm burden	Otero-Abad et al., 2017
Magnetic Capture RT-PCR	Moderate	−80 °C freezer, Tissue homogenizer, magnet, rotator, heat block, RT-PCR system	88.2 (79.8–93.9)	99.9 (99.7–100)	-Requires skilled technician -Throughput can be optimized using an automated nucleic acid extraction robot	Isaksson et al., 2014

Canids – serology						
EUROLINE®-WB (IgG, rEm18, rEm95, rEgAgB)	Moderate	SDS-PAGE electrophoresis machine, incubator, UV visualization	100 (74–100)	98 (91–100)	-Requires skilled technician -Commercially available; recombinant antigens widely available	Frey et al., 2017
Western Blot (EmVF)	Moderate	SDS-PAGE electrophoresis machine, incubator, UV visualization	100 (77–100)	100 (94–100)	-Requires skilled technician	Frey et al., 2017
ELISA (EmVF, Em2, rEm95, rEm18)	High	Incubator, ELISA plate washer, ELISA reader	EmVF: 100 (78–100) Em2: 79 (52–92) rEm18: 79 (52–92) rEm95: 100 (72–100)	EmVF: 85 (74–92) Em2: 97 (89–99) rEm18: 85 (74–92) rEm95: 100 (93–100)	-Requires skilled technician -Throughput can be optimized using an automated robot	Frey et al., 2017

Humans – serology						
Em2-DIGFA^f^	High	DIGFA test kit	77.8	97.1	-Commercially available -Rapid diagnostic test suitable for field conditions	Feng et al., 2010
Antibody Gold Immuno-chromatographic assay	High	Immunochromatographic test kit	97.5^g^	95.8	-Commercially available -Rapid diagnostic test suitable for field conditions	Gao et al., 2018

Environment – soil						
Taeniid egg recovery/RT-PCR	Low	−80 °C freezer, centrifuge, RT-PCR system	33–100	NR	-Requires skilled technician -Detection depends on egg recovery assay, DNA extraction technique and PCR primers	Umhang et al., 2017

a Low - <20 samples/technician/day; Moderate - 21–50 samples/technician/day; High - >50 samples/technician/day.

b Sensitivity.

c Specificity.

d Multiplex Polymerase Chain Reaction using Trachsel et al., 2007 primers

e Enzyme-linked Immunosorbent Assay.

f Dot Immunogold Filtration Assay.

g Other test characteristics: PLR: 23.0 (17.3–30.7); NLR: 0.03 (0.01–0.07); PPV: 78.2 (72.8–82.7); NPV: 99.6 (99.0–99.9); Accuracy: 96 (94.8–97.0).

Of the two human serological rapid diagnostic tests evaluated against abdominal ultrasound as a reference standard, the antibody gold immunochromatographic assay demonstrated higher sensitivity (97.8%) and specificity (95.8%) than the Em2-DIGFA assay. Both tests are commercially available, field stable, and can be used to rapidly screen large numbers of people (Table 6).

Six of the nine studies reported the analytical approach used to calculate sensitivity and specificity, and these were as follows: standard 2 × 2 calculation and modelling methods (Receiver Operating Curve/Area Under the Curve, Hui-Walter maximum likelihood estimation, and Bayesian Latent Class model; Iskenderali Ziadinov et al., 2008; Jiang et al., 2012; Otero-Abad et al., 2017; Gao et al., 2018; Isaksson et al., 2014; Frey et al., 2017). Ziadinov et al., 2008 used maximum likelihood estimation to determine test characteristics in dog populations from different villages in Kyrgyzstan, while Otero-Abad et al., 2017 employed Bayesian latent class analysis to jointly estimate test characteristics of four tests, prevalence and covariates related to prevalence in foxes. Both methods allow for determination of sensitivity and specificity in the absence of a gold standard. No studies were identified that evaluated new methods of detecting *E. multilocularis* in environmental samples at the Phase III field level.

### 3.6 QUADAS 2 quality appraisal

Only one of the nine studies that evaluated diagnostic assays for *E. multilocularis* surveillance in people, canids or the environment was considered low risk of bias and concern across all QUADAS2 criteria (Table 7; Gao et al., 2018). High risk of bias related to index and reference tests was generally a result of poor transparency on interpreting test thresholds and/or lack of clarity on whether diagnosticians were blinded between index and reference test results. The high risk of bias associated with participant selection was due to the frequent use of case-control studies and unclear reporting on exclusion criteria. Similarly, lack of information regarding time intervals between reference and index tests contributed to the assessment of high bias risk for flow and timing for approximately half of studies. In general, few applicability concerns were identified, indicating that participant selection and the use and interpretation of index and reference tests matched the questions posed by the systematic review.

**Table 7:** Quality appraisal of studies that reported diagnostic test characteristics of novel methods for *Echinococcus multilocularis* surveillance in people, canids, or the environment at the population level (2008–2018).

Title	Author, year	Risk of bias				Applicability concerns		
		Patient selection	Index test	Reference standard	Flow and timing	Patient selection	Index test	Reference standard
Dot immunogold filtration assay (DIGFA) with multiple native antigens for rapid serodiagnosis of human cystic and alveolar echinococcosis	Feng et al. 2010	●	●	○	○	○	○	○
Field evaluation of an immunochromatographic test for diagnosis of cystic and alveoloar Echinococcus	Gao et al. 2018	○	○	○	○	○	○	○
Dogs as victims of their own worms: serodiagnosis of canine alveolar echinococcosis	Frey et al. 2017	●	●	○	●	●	●	○
A semi-automated magnetic capture probe based DNA extraction and real-time PCR method applied in the Swedish surveillance of *Echinococcus multilocularis* in red fox (*Vulpes vulpes*) fecal samples	Isaksson et al. 2014	●	●	●	●	○	○	○
Specific detection of Echinococcus spp. from the Tibetan fox (*Vulpes ferrilata*) and the red fox (*V. vulpes*) using copro-DNA PCR analysis	Jiang et al. 2012	○	●	●	●	○	○	○
Latent class models for *Echinococcus multilocularis* diagnosis in foxes in Switzerland in the absence of a gold standard	Otero-Abad et al. 2017	●	●	○	○	○	○	○
Segmental Sedimentation and Counting Technique (SSCT): An adaptable method for qualitative diagnosis of Echinococcus multilocularis in fox intestines	Umhang et al. 2011	●	Δ	●	●	○	○	○
Canine echinococcosis in Kyrgyzstan: Using prevalence data adjusted for measurement error to develop transmission dynamics models	Ziadinov et al. 2008	○	●	○	○	○	○	○
Comparison of different commercial DNA extraction kits and PCR protocols for the detection of *Echinococcus multilocularis* eggs in fecal samples from foxes	Maksimov et al. 2017	●	●	○	Δ	●	○	○

● Not consistent with criteria, high risk of bias; ○ Consistent with criteria, low risk of bias; Δ Unknown risk of bias.

### 3.7 Grey literature search

Searches performed on grey literature search engines and government databases collected 276 reports, of which 12 were health system protocols or surveillance reports from endemic countries and deemed relevant (Table 8). Nine of these were European Union (EU) reports on surveillance methodology and outcomes in canids and humans, of which two provided information on diagnostic methods (Madslien et al., 2013; Antolova et al., 2014). Annual surveillance for *E. multilocularis* in red foxes in Norway is conducted to maintain official “free-from” status, for which the 2011–2012 assessment utilized magnetic capture real-time PCR to detect positive cases (Madslien et al., 2013). Antolova et al., 2014 carried out multi-year surveillance in Slovakia, using nested PCR for dogs, SCT for red foxes as well as ELISA, western blot and imaging techniques for humans (Table 8). While diagnostic techniques were not reported in EFSA surveillance reports, the 2015 citation states that the human case definition of echinococcosis does not differentiate between the two forms of the disease (EFSA, 2015). The EU defines human echinococcosis as at least one of the following: (1) histopathology or parasitology compatible with *E. multilocularis* OR *E. granulosus* (direct visualization of the protoscolex in cyst fluid); (2) detection of *E. granulosus* pathognomonic macroscopic morphology of cyst(s) in surgical specimens; (3) typical organ lesions detected by imaging techniques (CT, sonography or MRI) AND confirmed by a serological test; (4) *Echinococcus* spp. specific serum antibodies by high-sensitivity serological test AND confirmed by a high specificity serological test; (5) detection of *E. multilocularis* or *E. granulosus* nucleic acid in a clinical specimen (ECDC, 2018b). Three Canadian health system protocols were found, including two 2018 Ontario Public Health Standards outlining guidance on the natural history, transmission and public health management of *E. multilocularis* in humans (MOHLTC, 2018a; MOHLTC, 2018c). Appendix B provided provincial case definitions and appropriate diagnostic methods for case detection and surveillance, citing that serological testing for specimens collected in Ontario is analyzed using a combination of Em2-ELISA, II/3-10-ELISA or Em2Plus-ELISA at the Institute of Parasitology, University of Bern, Switzerland (MOHLTC, 2018a). The third report detailed public health guidance for sampling and detection of *E. multilocularis* in animals but did not provide details of testing methods (MOHLTC, 2018b).

**Table 8:** English language grey literature reports of country level surveillance or regulations pertaining to *Echinococcus multilocularis* in canids, humans, or environment (2008–2018).

Title	Author, year	Report type	Host/source	Case definition(s)	Reportable/notifiable	Diagnostic method(s)	Prevalence % (95%CI)
Scientific and technical assistance on *Echinococcus multilocularis* infection in animals	EFSA, 2012	Health system protocol	Canids	Any definitive host animal confirmed positive for *E. multilocularis* based on the results of the diagnostic tests described in Annex II of Regulation (EU) No 1152/2011 and having epidemiological information consistent with infection in the country	EU Regulation No 1152/2011	NA	NA
The surveillance and control programme for *Echinococcus multilocularis* in red foxes (*Vulpes vulpes*) in Norway. Hunting season 2011–2012.	Madslien et al., 2013	Annual surveillance report	Red foxes	RT-PCR +ve	NR	Magnetic Capture RT-PCR	0 (0–0.51)
Alveolar echinococcosis in a highly endemic area of northern Slovakia between 2000 and 2013	Antolova et al., 2014	Multi-year surveillance report	Dogs Red foxes Humans	Dogs, foxes: NR Humans: At least 2 of following 4 criteria: (i) presence of typical organ lesions, (ii) presence of antibodies to *E. multilocularis*, (iii) histological findings compatible with *E. multilocularis* metacestode, or (iv) presence of *E. multilocularis* DNA	NR	Dogs: Nested PCR Red foxes: SCT Humans: ELISA, Western blot, Imaging (Ultrasound, MRI, CT)	Dogs: 2.9 Red fox: 26–50 Humans: 26 cases
The European Union summary report on trends and sources of zoonoses, zoonotic agents and food-borne outbreaks in 2013	EFSA and ECDC, 2015	Annual surveillance report	Foxes (Human data reported in ECDC annual reports)	NR	Zoonoses Directive 2003/99/EC,	NR	Germany = 21.87 Slovakia = 22.3 Lux = 5.41 Sweden = 3.17
The European Union summary report on trends and sources of zoonoses, zoonotic agents and food-borne outbreaks in 2014	EFSA and ECDC, 2015	Annual surveillance report	Foxes (Human data reported in ECDC annual reports)	NR	Zoonoses Directive 2003/99/EC,	NR	Sweden 0.1 Denmark 2.0 Germany 23.4 Slovakia 15.8 Hungary 9.9
Annual epidemiological report 2014 - Echinococcosis	ECDC, 2016	Annual surveillance report	Humans	2008 or 2012^a^ Case definition acceptable.	NR	NR	82 cases from 7 EU/EEA countries
The European Union summary report on trends and sources of zoonoses, zoonotic agents and food-borne outbreaks in 2015	EFSA and ECDC, 2016	Annual Surveillance Report	Foxes (Human data reported in ECDC annual reports)	NR	Zoonoses Directive 2003/99/EC,	NR	Luxembourg 26.9 Switzerland 28.6 Germany 23.6 France 21.5 Slovakia 21.5 Denmark 8.06 Hungary 5.5 Sweden 0.1
Echinococcosis - Annual Epidemiological Report for 2015	ECDC, 2017	Annual Surveillance Report	Humans	2008 or 2012^a^ Case definition acceptable.	NR	NR	135 cases and 1 death from 6 countries (Table 2 lists countries for 2014 and 2015)
Echinococcosis - Annual Epidemiological Report for 2016	ECDC, 2018a	Annual Surveillance Report	Humans	2008 or 2012^a^ Case definition acceptable.	NR	NR	104 cases
Ministry of Health and Long-term Care Infectious Diseases Protocol, Appendix A, Chapter: *Echinococcus multilocularis* infection	MOHLTC, 2018a	Health system protocol	Humans	NR	Health Protection and Promotion Act, R.R.O. 1990, Reg. 569, Reports, (2018), and as per Requirement #3 of the “Reporting of Infectious Diseases” section of the Infectious Disease Protocol, 2018.	NR	NA
Ministry of Health and Long-term Care Infectious Diseases Protocol, Appendix B: Provincial case definitions for diseases of public health significance	MOHLTC, 2018b	Health system protocol	Humans	Ontario provincial case definition for human infection with *E. multilocularis* (in the presence of clinically compatible signs and symptoms): • Demonstration of antibodies to *E. multilocularis* in blood or serum sample OR • Demonstration of larval stages of *E. multilocularis* in histopathology samples from tissue biopsies	Confirmed and probable cases of disease are provincially reportable.	Serology performed at the Institute of Parasitology, University of Berne, (Switzerland): • Em2- ELISA • II/3-10- ELISA • Em2Plus-ELISA Confirmatory assays: PCR (tissue biopsies), direct immunofluorescence	NA
Management of *Echinococcus multilocularis* Infections in Animals Guideline, 2018	MOHLTC, 2018c	Health system protocol	NR	NR	Communicable Diseases Regulation (R.R.O. 1990, Reg. 557). A veterinarian or laboratory director who knows or suspects that one or more animals is infected with *E. multilocularis* shall notify the Medical Officer of Health within one business day. The board of health shall report all cases of *E. multilocularis* in animals to the ministry after receiving the report.	NR	NA

EFSA = European Food Safety Authority; ECDC = European Centers for Disease Control; OMHLTC = Ontario Ministry of Health and Long-term Care; NA = Not applicable; NR = Not reported, RT-PCR = Real Time Polymerase Chain Reaction.

a 2012 case definition for echinococcosis is at least one of the following five: (1) histopathology or parasitology compatible with *E. multilocularis* OR *E. granulosus* (direct visualization of the protoscolex in cyst fluid); (2) detection of *E. granulosus* pathognomonic macroscopic morphology of cyst(s) in surgical specimens; (3) typical organ lesions detected by imaging techniques (computerized tomography, sonography or MRI) AND confirmed by a serological test; (4) *Echinococcus* spp. specific serum antibodies by high-sensitivity serological test AND confirmed by a high specificity serological test; (5) detection of *E. multilocularis* or *E. granulosus* nucleic acid in a clinical specimen.

## 4 Discussion

Despite recent improvements to diagnostic technologies, AE remains a life-threatening infection for humans and animals; in part because patients across endemic countries do not have equal access to modern diagnostics and treatments. This systematic review identified examples of case finding in 27 of 43 countries where *E. multilocularis* is known to be present, confirming that there continue to be knowledge gaps in the global distribution and burden of this parasite (Deplazes et al., 2017; Torgerson et al., 2010; EFSA and ECDC, 2016). Prevalence estimates were most notably missing from Russia, which has the second highest reported annual incidence after China (Torgerson et al., 2010), and were also missing from other Central Asian countries (Azerbaijan, Georgia, Mongolia, Tadjikistan, Uzbekistan, Turkmenistan). Time period and language limitations of our search strategy might partially explain these gaps; however, it is also likely that limitations in reporting infrastructure, access and availability of diagnostic tests, poor physician and veterinarian awareness, long asymptomatic period, and presence of other closely related cestodes contribute to the general issues of under-diagnosis and under-reporting universal to characterizing this parasite (Eckert et al., 2001; EFSA and ECDC, 2016). These barriers to understanding the true burden of AE are cause for concern because *E. multilocularis* appears to be emerging in areas of North America, Europe and Asia.

This review identified nine protocols that were evaluated through Phase III and Phase III field trials for accuracy in diagnosing *AE*. There is currently no international consensus on specific gold standard protocols to detect this parasite in humans, animals, or the environment. Recommendations by the World Health Organization-Informal Working Group on Echinococcosis state that diagnostic criteria for AE in humans require parasite detection using at least one of the following: (i) imaging, (ii) serology, (iii) histopathology, (iv) nucleic acid detection (Brunetti et al., 2010). Many laboratories consider SCT to be the gold standard for case detection in wild canids (Eckert et al., 2001; Conraths and Deplazes, 2015), and as a result, new diagnostic techniques are often evaluated against this standard. However, two recent studies, one comparing SCT and three other diagnostic techniques in latent class models (Otero-Abad et al., 2017) and the second comparing SCT directly to magnetic capture RT-PCT (Isaksson et al., 2014), provide evidence that the sensitivity of SCT is lower than originally thought (Eckert et al., 2001). Therefore, it would be sensible to reconsider the value of SCT in surveillance and as a reference standard for diagnostic test evaluations (Conraths and Deplazes, 2015). As importantly, intestinal examination requires death of the animal, which has ecological effects when conducted as part of mass surveillance, and which is also not suitable for diagnosing infection in domestic or captive canids. We noted some studies conducting intestinal examination as a stand-alone determinant of *E. multilocularis* infection did not report morphologically identifying cestodes, which would delay early detection of other emerging *Echinococcus* species. Our study suggests that the latest ante-mortem developments in commercial and in-house technology lack diagnostic sensitivity and/or specificity (Table 5), although older techniques excluded by the timeline of this study do exist (EFSA and ECDC, 2016). Magnetic capture RT-PCR performed on fecal matter had the highest reported diagnostic accuracy of these assays and can be semi-automated for mass surveillance but requires costly equipment and reagents (Isaksson et al., 2014). In contrast, several promising serological tests were identified for detecting metacestode infection in human and canid hosts, each with sensitivity and sensitivity exceeding 95%. These included a commercially available but modified EUROLINE®-Western Blot (based on IgG, rEm18, rEm95, rEgAgB), an in-house Western Blot (EmVF) and an in-house ELISA (rEm95) for use in dogs, and a commercially available antibody gold immunochromatographic rapid diagnostic test for use in people (Gao et al., 2018; Frey et al., 2017). Diagnostic tests have differing capacity for detecting pre-patent, early metacestode and low intensity infections (Conraths and Deplazes, 2015). Furthermore Phase III field evaluations of test accuracy are often performed on one host in one locale, ignoring potential differences in accuracy across host species and prevalence (Conraths and Deplazes, 2015). Therefore, mass screening campaigns should consider the epidemiological situation of a region and the detection limits of diagnostic tests when creating a surveillance strategy.

Laboratory capacity is an integral component of health system infrastructure, and such services play a key role in detection, assessment, response, notification, and monitoring of public health events. According to the World Bank, seven AE endemic countries were classified as low income (Kyrgyzstan, Tadjikistan) or low-middle income (Georgia, Mongolia, Uzbekistan, Moldova, Ukraine) at the mid-point of this review; of these, our search only captured prevalence estimates for Kyrgyzstan (The World Bank, 2018; Ziadinov et al., 2008; Ziadinov et al., 2010). Some *E. multilocularis* detection techniques, such as SCT, arecoline hydrobromide purgation and Em2-DIGFA, require minimal equipment and are feasible for a range of settings. However, most molecular and immunological detection techniques require significant investments to laboratory infrastructure and technician training as well as access to expensive reagents, making them inaccessible to diagnosticians in low resource regions. It is also important to consider the safety aspects of various diagnostic tests. For example, dogs treated with arecoline hydrobromide have died of bone splinters puncturing the intestinal tract. Technicians collecting and analyzing freshly purged fecal matter must be properly equipped with personal protective equipment, and environments housing purged animals must be thoroughly decontaminated from infectious *E. multilocularis* eggs (Eckert et al., 2001). High income countries currently engaged in *E. multilocularis* surveillance can play a greater role in building capacity for surveillance, diagnostics, research and treatment among lower resource countries, which would ultimately deliver mutual benefits given the ability of infected wild canid to move freely between endemic and non-endemic regions. Moreover, it is not only low income countries experiencing capacity shortages. The outsourcing of Ontario medical diagnostics to Swiss laboratories for confirmatory testing suggests the need for improved laboratory capacity in Canada (MOHLTC, 2018c). As well, *E. multilocularis* specimens collected from humans do not appear to be sequenced routinely (our study found only one example (Ma et al., 2015)). This represents a lost opportunity to explore parasite origin, to confirm the emergence of specific haplotypes into new areas, or to investigate the biological and clinical significance of parasite diversity.

While our findings show Europe as a leading region in AE surveillance and reporting, diagnostic methods were not explicitly stated within ECDC reports, likely due to the lack of standardization across Member State (MS) laboratories (EFSA Panel on Animal Health and Welfare, 2015). Moreover, there exists a high degree of discrepancy of diagnostic test characteristics reported between MS, and as reported by pooled meta-analysis evaluations (Conraths and Deplazes, 2015; EFSA Panel on Animal Health and Welfare, 2015; Casulli et al., 2015). One third of diagnostic accuracy evaluations in our study did not report how sensitivity and specificity were calculated, while others utilized advanced modelling methods to compare measures across prevalence parameters. These findings indicate the need for standardized internal AE protocols for laboratory diagnostics within endemic and newly emerging regions. Our team did not carry out meta-analysis for test characteristics given the small number of studies collected and the variation of test types, host populations and population prevalence. We chose not to report pooled estimates of population prevalence, as combining studies by higher-level geographical designations would misrepresent prevalence variations by regions.

These diagnostic considerations bear strongly on mechanisms of disease reporting, and particularly on case definitions for surveillance. Definitions utilized by the EU and Ontario (Canada) denote a case positive by the outcome of any one of a number of tests. Moreover, some components of the case definition algorithms require a positive result from multiple diagnostic tests run in series to yield a final positive classification. In our study, case definitions in primary surveillance studies were often not described, and it was difficult to interpret the relationships between multiple tests in a diagnostic procedure. The reliance of case definitions on multiple test outcomes, combined with substantial variation in test characteristics, predictive values and performance of gold standards, is a barrier to accurately assessing the confidence of reported prevalence estimates. This is further compounded by a lack of consistency in application of case definitions within health jurisdictions. For example, while the EU has an established annual reporting system for *E. multilocularis*, only 23 countries in 2016 reported echinococcosis cases using the 2008 or 2012 case definitions, neither of which are species specific (ECDC, 2018b). Species specific reporting is especially important for co-endemic regions, and is also a problem in Canada, where patient records obtained from the Canadian Institute for Health Information show that physicians often do not differentiate between *Echinococcus* species (Schurer et al., 2015). Development of case definitions should consider the test characteristics of diagnostic procedures wherever possible, be standardized for classifying/counting cases consistently across reporting jurisdictions, and should, at minimum, differentiate between *Echinococcus* species.

Our literature search did not identify a source that summarized mandatory versus voluntary *E. multilocularis* reporting all AE endemic countries. In the EU, notification of human echinococosis is mandatory for 22 MS, although other countries do voluntarily report cases (EFSA and ECDC, 2016). Echinococcosis in animals is notifiable in 17 MS, and contamination of food is notifiable in 10 MS (EFSA and ECDC, 2016). Surveillance for *E. multilocularis* in Europe is usually carried out on red foxes, and is predominantly diagnosed using morphological (SCT) or molecular (PCR) methods. Four EU countries (Finland, Ireland, Malta and the United Kingdom) are considered free from *E. multilocularis* and must conduct annual surveillance to retain this status (as per Regulation (EU) No 1152/201117). Human AE is not nationally notifiable in Canada, but it became provincially reportable in 2018 in response to heightened prevalence in wild canids and the novel detection of AE in domestic dogs within Ontario (Government of Ontario, 2018). Detection of *E. multilocularis* in animals and food is not currently notifiable and we did not find any documents to suggest that the government was involved in active surveillance in people, animals, or food. Human AE is reportable in China (Ding and Li, 2016), Turkey (Altintas, 2008) and Kyrgyzstan (Usubalieva et al., 2013). Considering the increased concern for this parasite in regions of Canada, Europe and Asia, there exists a need for mandatory reporting frameworks based on consistent case definitions. Moreover, developers of surveillance and reporting frameworks should consider applying a One Health approach to create enhanced systems that work synergistically in monitoring human, animal and environmental sources, especially as this parasite continues to build in importance.

### 4.1 Conclusions

Individuals infected with AE require early, affordable, and accurate diagnosis as well as access to modern treatment to ensure a favourable prognosis. Our study identified barriers to this goal that included scarce surveillance in low and low-middle income AE endemic countries, lack of consensus on diagnostic gold standards, reliance on convenience sampling for human and canid studies, poor reporting of case definitions, genus versus species level diagnosis, and infrequent submission of nucleotide sequences public databases. Improving reporting infrastructure systems is an important next step to comprehensively defining the global health burden and geographic distribution of *E. multilocularis*, as well as to monitoring emergence of this parasite into new areas and host types. Mandatory reporting of human cases in endemic countries and animal cases in non-endemic countries, data sharing between government agencies engaged in human, animal and environmental surveillance, and strengthened partnerships between low and high resource countries are all strategies to optimize health equity for AE patients. Furthermore, national *E. multilocularis* control programs should consider diagnostic test limitations with respect to host species, estimated local prevalence, and presence of other *Echinococcus* species when designing surveillance strategies. The formation of a joint WHO/OIE world reference laboratory with a mandate to develop diagnostic protocols, identify *Echinococcus* species, and design integrated human-animal surveillance systems could go a long way in achieving these goals.

## Appendix A Supplementary data

Supplementary references Image 1
