# Supplementary material for: A One Health systematic review of diagnostic tools for Echinococcus multilocularis surveillance: Towards equity in global detection
Source: Food Waterborne Parasitol. 2019 Apr 16;15:e00048. doi: 10.1016/j.fawpar.2019.e00048 (PMC7034026; doi:10.1016/j.fawpar.2019.e00048)
Supplement: Supplementary file 1 — Supplementary references [file mmc1.doc]

**Supplementary References**

Antolova, D., Reiterova, K., Miterpakova, M., Dinkel, A., and Dubinsky, P. (2009). The First Finding of *Echinococcus multilocularis* in Dogs in Slovakia: An Emerging Risk for Spreading of Infection. Zoonoses and Public Health. 56(2), 53-58. <https://doi.org/10.1111/j.1863-2378.2008.01154.x>.

Antolova, D., Miterpakova, M., Radoňak, J., Hudačkova, D., Szilagyiova, M., and Začek, M. (2014). Alveolar Echinococcosis in a Highly Endemic Area of Northern Slovakia between 2000 and 2013. Euro Surveillance: European Communicable Disease Bulletin. 19(34), 1-8.

Bagrade, G., Šnábel, V., Romig, T., Ozoliņš, J., Hüttner, M., Miterpáková, M., Ševcová, D., and Dubinskỳ, P. (2008). *Echinococcus multilocularis* Is a Frequent Parasite of Red Foxes (*Vulpes vulpes*) in Latvia. Helminthologia. 45 (4), 157–161.

Bagrade, G., Kirjusina, M., Vismanis, K., and Ozoliņs, J. (2009). Helminth Parasites of the Wolf *Canis lupus* from Latvia. Journal of Helminthology. 83 (1), 63–68. <https://doi.org/10.1017/S0022149X08123860>.

Bagrade, G., Deksne, G., Ozoliņa, Z., Howlett, SJ., Interisano, M., Casulli, A., and Pozio, E. (2016). *Echinococcus multilocularis* in Foxes and Raccoon Dogs: An Increasing Concern for Baltic Countries. Parasites & Vectors. 9(1), 615.

Barabási, SS., Deplazes, P., Cozma, V., Pop, S., Tivadar, C., Bogolin, I., Popescu, R. *et al*. (2010). *Echinococcus multilocularis* Confirmed in Romania. Sci. Parasitol. 11, 89–96.

Beiromvand, M., Rafiei, A., Razmjou, E., and Maraghi, S. (2018). Multiple Zoonotic Helminth Infections in Domestic Dogs in a Rural Area of Khuzestan Province in Iran. BMC Veterinary Research. 14(1):224. <https://doi.org/10.1186/s12917-018-1529-6>.

Beiromvand, M., Akhlaghi, L., Massom, SHF., Mobedi, I., Meamar, AR., Oormazdi, H., Motevalian, A., and Razmjou, E. (2011). Detection of *Echinococcus multilocularis* in Carnivores in Razavi Khorasan Province, Iran Using Mitochondrial DNA. PLoS Neglected Tropical Diseases. 5(11), e1379. <https://doi.org/10.1371/journal.pntd.0001379>.

Borecka, A., Gawor, J., Malczewska, M., and Malczewski, A. (2008). Occurrence of *Echinococcus multilocularis* in Red Foxes (*Vulpes vulpes*) in Southern Poland. Helminthologia 45 (1): 24–27.

Bruzinskaite, R., Sarkunas, M., Torgerson, P, Mathis, A., and Deplazes, P. (2009). Echinococcosis in Pigs and Intestinal Infection with Echinococcus Spp. in Dogs in Southwestern Lithuania. Veterinary Parasitology. 160(3-4), 237-241. <https://doi.org/10.1016/j.vetpar.2008.11.011>.

Cadavid Restrepo, AM., Yang, YR., McManus, DP., Gray, DJ., Barnes, TS., Williams, GM., Soares Magalhães, RJ., and Clements, ACA. (2018). Environmental Risk Factors and Changing Spatial Patterns of Human Seropositivity for Echinococcus Spp. in Xiji County, Ningxia Hui Autonomous Region, China. Parasites & Vectors. 11(1), 159. <https://doi.org/10.1186/s13071-018-2764-1>.

Cai, H., Guan, Y. Ma, X. Wang, L., Wang, H., Su, G., Zhang, X. *et al*. (2017). Epidemiology of Echinococcosis Among Schoolchildren in Golog Tibetan Autonomous Prefecture, Qinghai, China. The American Journal of Tropical Medicine and Hygiene. 96(3), 674–79. <https://doi.org/10.4269/ajtmh.16-0479>.

Casulli, A., Szell, Z., Pozio, E., and Sreter, T. (2010). Spatial Distribution and Genetic Diversity of *Echinococcus multilocularis* in Hungary. Veterinary Parasitology. 174(3-4), 241-246. <https://doi.org/10.1016/j.vetpar.2010.08.023>.

Catalano, S., Lejeune, M., Liccioli, S., Verocai, GG., Gesy, KM., Jenkins, EJ., Kutz, SJ., Fuentealba, C ., Duignan, PJ., and Massolo, A. (2012). *Echinococcus multilocularis* in Urban Coyotes, Alberta, Canada. Emerging Infectious Diseases. 18(10): 1625-1628. <http://dx.doi.org/10.3201/eid1810.120119>.

Cisak, E., Sroka, J., Wojcik-Fatla, A., Zajac, V., and Dutkiewicz, J. (2015). Evaluation of Reactivity to Echinococcus Spp. among Rural Inhabitants in Poland. Acta Parasitologica. 60(3), 525-529. <https://doi.org/10.1515/ap-2015-0074>.

Comte, S., Raton, V., Raoul, F., Boue, F., Umhang, G., Favier, S., Dunoyer, C., Woronoff, N., Giraudoux, P., and Combes, B. (2012). Westward Spread of *Echinococcus multilocularis* in Foxes, France, 2005-2010. Emerging Infectious Diseases.18(12), 2059-2062. <https://doi.org/10.3201/eid1812.120219>.

Comte, S., Raton, V., Raoul, F., Hegglin, D., Giraudoux, P., Deplazes, P., Favier, S., *et al*. (2013). Fox Baiting against *Echinococcus multilocularis*: Contrasted Achievements among Two Medium Size Cities. Preventive Veterinary Medicine. 111 (1–2), 147–55. <https://doi.org/10.1016/j.prevetmed.2013.03.016>.

Comte, S., Umhang, G., Raton, V., Raoul, F., Giraudoux, P., Combes, B., and Boué, F. (2017). *Echinococcus multilocularis* Management by Fox Culling: An Inappropriate Paradigm. Preventive Veterinary Medicine. 147, 178–85. <https://doi.org/10.1016/j.prevetmed.2017.09.010>.

Denzin, N., Schliephake, A., Fröhlich, A., Ziller, M. and Conraths, FJ. (2014). On the Move? *Echinococcus multilocularis* in Red Foxes of Saxony-Anhalt (Germany). Transboundary and Emerging Diseases. 61(3), 239–46. <https://doi.org/10.1111/tbed.12026>.

Dyachenko, V., Pantchev, N., Gawlowska, S., Vrhovec, MG., and Bauer, C. (2008). *Echinococcus multilocularis* Infections in Domestic Dogs and Cats from Germany and Other European Countries. Veterinary Parasitology. 157(3-4), 244-253. <https://doi.org/10.1016/j.vetpar.2008.07.030>.

European Centre for Disease Prevention and Control. (2016). Echinococcosis. Annual Epidemiological Report for 2014. ECDC. Available at: <https://ecdc.europa.eu/sites/portal/files/documents/AER_for_2015-echinococcosis.pdf> . Last accessed: 4Mar2019.

European Centre for Disease Prevention and Control. (2017). Echinococcosis. Annual Epidemiological Report for 2015. ECDC. Available at: <https://ecdc.europa.eu/sites/portal/files/documents/AER_for_2015-echinococcosis.pdf> . Last accessed: 4Mar2019.

European Centre for Disease Prevention and Control. (2018). Echinococcosis. Annual Epidemiological Report for 2016. ECDC. Available at: <https://ecdc.europa.eu/sites/portal/files/documents/AER_for_2016-echinococcosis.pdf>. Last accessed: 4Mar2019.

EFSA and ECDC (European Food Safety Authority and European Centre for Disease Prevention and Control). (2015). The European Union Summary Report on Trends and Sources of Zoonoses, Zoonotic Agents and Foodborne Outbreaks in 2013. EFSA Journal. 13(1), 165.

EFSA and ECDC (European Food Safety Authority and European Centre for Disease Prevention and Control). (2015). The European Union Summary Report on Trends and Sources of Zoonoses, Zoonotic Agents and Foodborne Outbreaks in 2014. EFSA Journal. 13(12), 4329.

EFSA and ECDC (European Food Safety Authority and European Centre for Disease Prevention and Control). (2016). The European Union Summary Report on Trends and Sources of Zoonoses, Zoonotic Agents and Foodborne Outbreaks in 2015. EFSA Journal. 13(12), 4329.

European Food Safety Authority. (2012). Scientific and technical assistance on *Echinococcus multilocularis* infection in animals. EFSA Journal. 10(11), 2973.

Feng, X., Wen, H., Zhang, Z., Chen, X., Ma, X., Zhang, J., Qi, X., Bradshaw, H., Vuitton, D., and Craig, PS. (2010). Dot Immunogold Filtration Assay (DIGFA) with Multiple Native Antigens for Rapid Serodiagnosis of Human Cystic and Alveolar Echinococcosis. Acta Tropica. 113(2), 114–120.

Frey, CF., Marreros, N., Renneker, S., Schmidt, L., Sager, H., Hentrich, B., Milesi, S., and Gottstein, B. (2017). Dogs as Victims of Their Own Worms: Serodiagnosis of Canine Alveolar Echinococcosis. Parasites and Vectors. 10(422), 1-8. <https://doi.org/10.1186/s13071-017-2369-0>.

Gao, C., Wang, JY., Shi, F., Steverding, D., Wang, X., Yang, YT., and Zhou, XN. (2018). Field Evaluation of an Immunochromatographic Test for Diagnosis of Cystic and Alveolar Echinococcosis. Parasites & Vectors 11 (1): 311. <https://doi.org/10.1186/s13071-018-2896-3>.

Gesy, KM., Schurer, JM., Massolo, A., Liccioli, S., Elkin, BT., Alisauskas, R., and Jenkins, EJ. (2014). Unexpected Diversity of the Cestode *Echinococcus multilocularis* in Wildlife in Canada. International Journal for Parasitology: Parasites and Wildlife 3: 81–87.

Gesy, KM, Schwantje, H., Liccioli, S., and Jenkins, EJ. (2013). Establishment of a European-Type Strain of *Echinococcus multilocularis* in Canadian Wildlife. Parasitology 140: 1133–37.

Gurler, AT., Gori, F., Bolukbas, CS., Umur, S., Acici, M., and Deplazes, P. (2018). Investigation of *Echinococcus multilocularis* in Environmental Definitive Hosts Feces in the Asian and the European Parts of Turkey. Front Vet Sci. 15(5), 48.

Guislain, MH., Raoul, F., Giraudoux, P., Terrier, ME., Froment, G., Ferte, H., and Poulle, ML. (2008). Ecological and Biological Factors Involved in the Transmission of *Echinococcus multilocularis* in the French Ardennes. Journal of Helminthology. 82(2), 143-151. <https://doi.org/10.1017/S0022149X08912384>.

韩秀敏,王虎,蔡辉霞,马霄,刘玉芳,韦炳辉， Ito A， Craig PS. (2009). 青海省达日县棘球蚴病流行病学调查. *中国寄生虫学与寄生虫病杂志,* 27: 5-26. [Han X, Wang H, Cai H, Ma X, Liu Y, Wei B, Ito A, Craig PS. Epidemiological survey on echinococcosis in Darlag County of Qinghai Province. Chin J Parasitol Parasit Dis. 2009; 27:22-26.]

Han, J., Bao, G., Zhang, D., Gao, P., Wu, T., Craig, P., Giraudoux, P. *et al*. (2015). A Newly Discovered Epidemic Area of *Echinococcus multilocularis* in West Gansu Province in China. PloS One 10 (7): e0132731. <https://doi.org/10.1371/journal.pone.0132731>.

韩秀敏,张学勇,蔡其刚,张静妮,王永顺,张强.(2017). 青海省南部高原藏族儿童泡型包虫病流行现状分析. 中国血吸虫病防治杂志, 29:53-58. [Han X, Zhang X, Cai Q, Zhang J, Wang Y, Zhang Q. Epidemic status of alveolar echinococcosis in Tibetan children in south Qinghai Province. Chin J Schisto Control. 2017; 29:53-58.]

Han, XM., Cai, QG., Wang, W., Wang, H., Zhang, Q., and Wang, YS. (2018). Childhood Suffering: Hyper Endemic Echinococcosis in Qinghai-Tibetan Primary School Students, China. Infectious Diseases of Poverty 7 (1): 71. <https://doi.org/10.1186/s40249-018-0455-y>.

Hanosset, R, Saegerman, C., Adant, S., Massart, L, and Losson, B. (2008). *Echinococcus multilocularis* in Belgium: Prevalence in Red Foxes (*Vulpes vulpes*) and in Different Species of Potential Intermediate Hosts. Veterinary Parasitology 151 (2–4): 212–217.

Hermosilla, C., Kleinertz, S., Silva, LMR., Hirzmann, J., Huber, D., Kusak, J., and Taubert, A. (2017). Protozoan and Helminth Parasite Fauna of Free-Living Croatian Wild Wolves (*Canis lupus*) Analyzed by Scat Collection. Veterinary Parasitology. 233, 14-19. https://doi.org/10.1016/j.vetpar.2016.11.011.

Hurnikova, Z., Miterpakova, M., and Chovancova, B. (2009). The Important Zoonoses in the Protected Areas of the Tatra National Park (TANAP). Wiadomosci Parazytologiczne. 55(4), 395-398.

Isaksson, M., AAsa Hagström, A., Armua-Fernandez, MT., Wahlström, H., AAgren, EO., Miller, A., Holmberg, A. *et al*. (2014). A Semi-Automated Magnetic Capture Probe Based DNA Extraction and Real-Time PCR Method Applied in the Swedish Surveillance of *Echinococcus multilocularis* in Red Fox (*Vulpes vulpes*) Faecal Samples. Parasites & Vectors. 7 (1), 583.

Karamon, J., Sroka, J., Cencek, T., Michalski, MM., Zięba, P. and Jacek Karwacki, J. (2011). Prevalence of *Echinococcus multilocularis* in Red Foxes in Two Eastern Provinces of Poland. Bull Vet Inst Pulawy. 55, 429–433.

Knapp, J., Giraudoux, P., Combes, B., Umhang, G., Boué, F., Said-Ali, Z., Aknouche, S., *et al*. (2018). Rural and Urban Distribution of Wild and Domestic Carnivore Stools in the Context of *Echinococcus multilocularis* Environmental Exposure. International Journal for Parasitology. 48(12), 937-946. <https://doi.org/10.1016/j.ijpara.2018.05.007>.

Melotti, JR., Muzzall, PM., O’Brien, DJ., Cooley, TM., and Tsao, JI. (2015). Low Prevalence of *Echinococcus multilocularis* in Michigan, USA: A Survey of Coyotes (*Canis latrans*), Red Foxes (*Vulpes vulpes*), and Gray Foxes (*Urocyon cinereoargenteus*), 2009–2012. Comparative Parasitology 82 (2): 285–290.

MOHLTC (2018). Ministry of Health and Long-term Care Infectious Diseases Protocol, Appendix A: Chapter - *Echinococcus multilocularis* infection. Available at: <http://www.health.gov.on.ca/en/pro/programs/publichealth/oph_standards/docs/E_multilocularis_chapter.pdf> . Last accessed: 4Mar2019.

MOHLTC (2018a). Ministry of Health and Long-term Care Infectious Diseases Protocol, Appendix B: Provincial case definitions for diseases of public health significance. Available at: <http://www.health.gov.on.ca/en/pro/programs/publichealth/oph_standards/docs/E_multilocularis_cd.pdf> . Last accessed: 4Mar2019.

MOHLTC (2018b). Management of *Echinococcus multilocularis* infections in animals guideline. Available at: <http://health.gov.on.ca/en/pro/programs/publichealth/oph_standards/docs/protocols_guidelines/Management_of_EM_Infections_in_Animals_2018.pdf>. Last accessed: 4Mar2019.

Takumi, K., de Vries, A., Chu, ML., Mulder, J., Teunis, P., and van der Giessen, J. (2008). Evidence for an Increasing Presence of *Echinococcus multilocularis* in Foxes in The Netherlands. Journal for Parasitology, 38(5), 571-578. <https://doi.org/10.1016/j.ijpara.2007.09.014>.

Karamon, J., Kochanowski, M., Sroka, J., Cencek, T., Rozycki, M., Chmurzynska, E., and Bilska-Zajac, E. (2014). The Prevalence of *Echinococcus multilocularis* in Red Foxes in Poland - Current Results (2009-2013). Parasitology Research,. 113(1), 317-22. <https://doi.org/10.1007/s00436-013-3657-z>.

Karamon, J., Kochanowski, M., Dąbrowska, J., Sroka, J., Różycki, M., Bilska-Zając, E., and Cencek, T. (2015). Dynamics of *Echinococcus multilocularis* Infection in Red Fox Populations with High and Low Prevalence of This Parasite in Poland (2007–2014). Bulletin of the Veterinary Institute in Pulawy. 59 (2), 213–217.

Karamon, J., Samorek-Pieróg, M., Kochanowski, M., Dabrowska, J., Sroka, J., Golab, E., Umhang, G., and Cencek, T. (2016). First Detection of *Echinococcus multilocularis* in Dogs in a Highly Endemic Area of Poland. Folia Parasitologica. 63: 1.

Kohansal, MH., Nourian, A., Haniloo, A., and Fazaeli, A. (2017). Molecular Detection of Taenia Spp. in Dogs’ Feces in Zanjan Province, Northwest of Iran. Veterinary World. 10(4), 445-449. <https://doi.org/10.14202/vetworld.2017.445-449>.

Jiang, W., Liu, N., Zhang, G., Renqing, P., Xie, F., Li, T., Wang, Z., and Wang, X. (2012). Specific Detection of Echinococcus Spp. from the Tibetan Fox (*Vulpes ferrilata*) and the Red Fox (*V. vulpes*) Using Copro-DNA PCR Analysis. Parasitology Research. 111 (4), 1531–39. <https://doi.org/10.1007/s00436-012-2993-8>.

Lass, A., Szostakowska, B., Myjak, P., and Korzeniewski, K. (2015). The First Detection of *Echinococcus multilocularis* DNA in Environmental Fruit, Vegetable, and Mushroom Samples Using Nested PCR. Parasitology Research. 114(111), 4023-4029. <https://doi.org/10.1007/s00436-015-4630-9>.

Lass, A., Szostakowska, B., Myjak, P., and Korzeniewski, K. (2017). Detection of *Echinococcus multilocularis* DNA in Fruit, Vegetable, and Mushroom Samples Collected in the Non-Endemic Territory of the Pomerania Province and Comparison of the Results with Data from Rural Areas of the Neighbouring Highly Endemic Warmia-Masuria Province, Poland. Acta Parasitologica. 62(2), 459-465. <https://doi.org/10.1515/ap-2017-0053>.

Laurimaa, L., Moks, E., Soe, E., Valdmann, H., and Saarma, U. (2016). *Echinococcus multilocularis* and Other Zoonotic Parasites in Red Foxes in Estonia. Parasitology. 143(11), 1450–58. <https://doi.org/10.1017/S0031182016001013>.

Laurimaa, L., Süld, K., Moks, E., Valdmann, H., Umhang, G., Knapp, J. and Saarma, U. (2015). First Report of the Zoonotic Tapeworm *Echinococcus multilocularis* in Raccoon Dogs in Estonia, and Comparisons with Other Countries in Europe. Veterinary Parasitology. 212(3–4), 200–205. <https://doi.org/10.1016/j.vetpar.2015.06.004>.

Li T, Chen, X., Zhen, R., Qiu, J., Qiu, D., Xiao, N., Ito, A., *et al*. (2010). Widespread Co-Endemicity of Human Cystic and Alveolar Echinococcosis on the Eastern Tibetan Plateau, Northwest Sichuan/Southeast Qinghai, China. Acta Tropica. 113(3), 248-256. <https://doi.org/10.1016/j.actatropica.2009.11.006>.

Li, W., Guo, Z., Duo, H., Fu, Y., Peng, M., Shen, X., Tsukada, H., *et al*. (2013). Survey on Helminths in the Small Intestine of Wild Foxes in Qinghai, China. Journal of Veterinary Medical Science. 75(1), 1329-1333. <https://doi.org/10.1292/jvms.13-0187>.

Liccioli, S., Kutz, SJ., Ruckstuhl, KE., and Massolo, A. (2014). Spatial Heterogeneity and Temporal Variations in *Echinococcus multilocularis* Infections in Wild Hosts in a North American Urban Setting. Journal for Parasitology. 44(7), 457-465 <https://doi.org/10.1016/j.ijpara.2014.03.007>.

刘灿. (2014). *宁夏包虫病时间地域分布调查*. (宁夏医科大学). [Liu C. Spatial-temporal investigation of echinococcosis in Ningxia. 2014. Ningxia Medical University, Yinchuan, Ningxia, China.]

Liu, CN., Xu, YY., Cadavid-Restrepo, AM., Lou, ZZ., Yan, HB., Li, L., Fu, BQ., *et al*. (2018). Estimating the Prevalence of Echinococcus in Domestic Dogs in Highly Endemic for Echinococcosis. Infectious Diseases of Poverty. 77(1), 77. <https://doi.org/10.1186/s40249-018-0458-8>.

马增光. (2014). *新疆昭苏盆地野生宿主多房棘球蚴病的流行病学调查*. (Doctoral dissertation, 新疆农业大学).[Ma Z. Epidemiological investigation of wild hosts of alveolar echinococcosis at Zhaosu Basin, Xinjiang. 2014. Xinjiang Agricultural University, Wulumuqi, Xinjiang, China.]

Ma, J., Wang, H., Lin, G., Zhao, F., Li, C., Zhang, T., Ma, X. *et al*. (2015). Surveillance of Echinococcus Isolates from Qinghai, China. Veterinary Parasitology. 207(1–2), 44–48. <https://doi.org/10.1016/j.vetpar.2014.11.012>.

Maas, M., Dam-Deisz WD., van Roon AM., Takumi, K., and van der Giessen, JW. (2014). Significant Increase of *Echinococcus multilocularis* Prevalence in Foxes, but No Increased Predicted Risk for Humans. Veterinary Parasitology. 206(3-4), 167-1772. <https://doi.org/10.1016/j.vetpar.2014.10.006>.

Maksimov, P., Schares, G., Press, S., Fröhlich, A., Basso, W., Herzig, M., and Conraths, FJ. (2017). Comparison of Different Commercial DNA Extraction Kits and PCR Protocols for the Detection of *Echinococcus multilocularis* Eggs in Faecal Samples from Foxes. Veterinary Parasitology. 237, 83–93. <https://doi.org/10.1016/j.vetpar.2017.02.015>.

Malczewski, A., Gawor, J., and Malczewska, M. (2008). Infection of Red Foxes (*Vulpes vulpes*) with *Echinococcus multilocularis* during the Years 2001-2004 in Poland. Parasitology Research. 103(3), 501-505 <https://doi.org/10.1007/s00436-008-0990-8>.

Massolo, A., Liccioli, S., Budke, CM., and Klein, C. (2014). *Echinococcus multilocularis* in North America: The Great Unknown. Parasite. 21: 73.

Miller, AL., Olsson, GE., Sollenberg, S., Skarin, M., Wahlström, H., and Höglund, J. (2016). Support for Targeted Sampling of Red Fox (*Vulpes vulpes*) Feces in Sweden: A Method to Improve the Probability of Finding *Echinococcus multilocularis*. Parasites & Vectors. 9(1), 613. <https://doi.org/10.1186/s13071-016-1897-3>.

Miterapakova, M. and Dubinsky, P. 2011. Fox tapeworm (*Echinococcus multilocularis*) in Slovakia – summarizing the long-term monitoring. Helminthologia. 48:155.

Mobedi, I., M. Zare-Bidaki, M., Siavashi, M., Naddaf, S., Kia, E., and Mahmoudi, M. (2013). Differential Detection of Echinococcus Spp. Copro-DNA by Nested-PCR in Domestic and Wild Definitive Hosts in Moghan Plain, Iran. Iranian Journal of Parasitology. 8(1): 107–13.

Moss JE., Chen, X., Li, T., Qiu, J., Wang, Q., Giraudoux, P., Ito, A., Torgerson, PR., and Craig, PS. (2013). Reinfection Studies of Canine Echinococcosis and Role of Dogs in Transmission of *Echinococcus multilocularis* in Tibetan Communities, Sichuan, China. Parasitology. 140(13), 1685-1692. https://doi.org/10.1017/S0031182013001200.

Nonaka, N., Kamiya, M., Kobayashi, F., Ganzorig, S., Ando, S., Yagi, K., Iwaki, T., Inoue, T., and Oku, Y. (2009). *Echinococcus multilocularis* Infection in Pet Dogs in Japan. Vector-Borne and Zoonotic Diseases. 9(2), 201-206. https://doi.org/10.1089/vbz.2008.0097.

Nonaka, N., Sano, T., Inoue, T., Armua, MT., Fukui, D., Katakura, K., and Oku, Y. (2009). Multiplex PCR System for Identifying the Carnivore Origins of Faeces for an Epidemiological Study on *Echinococcus multilocularis* in Hokkaido, Japan. Parasitology Research. 106, 75-83. https://doi.org/10.1007/s00436-009-1629-0.

Otero-Abad, B., Armua-Fernandez, MT., Deplazes, P., Torgerson, PR., and Hartnack, S. (2017). Latent Class Models for *Echinococcus multilocularis* Diagnosis in Foxes in Switzerland in the Absence of a Gold Standard. Parasites & Vectors. 10(1), 612. https://doi.org/10.1186/s13071-017-2562-1.

Petersen, HH., Al-Sabi, MNS., Enemark, HL., Kapel, CMO., Jørgensen, JA., and Chriél, M. (2018). *Echinococcus multilocularis* in Denmark 2012–2015: High Local Prevalence in Red Foxes. Parasitology Research. 117(8), 2577-2584.

Poeppl, W., Herkner, H., Tobudic, S., Faas, A., Mooseder, G., Burgmann, H., and Auer, H. (2013). Exposure to *Echinococcus multilocularis*, *Toxocara canis*, and *Toxocara cati* in Austria: A Nationwide Cross-Sectional Seroprevalence Study. Vector-Borne and Zoonotic Diseases. 13(11). 798–803.

Poulle, ML., Bastien, M., Richard, Y., Josse-Dupuis, E., Aubert, D., Villena, I., and Knapp, J. (2017). Detection of *Echinococcus multilocularis* and Other Foodborne Parasites in Fox, Cat and Dog Faeces Collected in Kitchen Gardens in a Highly Endemic Area for Alveolar Echinococcosis. Parasite. (24), 29. 1-11. https://doi.org/10.1051/parasite/2017031.

Robardet, E., Giraudoux, P., Caillot, C., Boue, F., Cliquet, F., Augot, D., and Barrat, J. (2008). Infection of Foxes by *Echinococcocus multilocularis* in Urban and Suburban Areas of Nancy, France: Influence of Feeding Habits and Environment. Parasite. 15(1), 77-85.

Schurer, Janna M., Emilie Bouchard, Ann Bryant, Sarah Revell, Grace Chavis, Anne Lichtenwalner, and Emily J. Jenkins. 2018. Echinococcus in Wild Canids in Québec (Canada) and Maine (USA). PLoS Neglected Tropical Diseases 12 (8): e0006712. https://doi.org/10.1371/journal.pntd.0006712.

Schurer, JM, BT Elkin, and EJ Jenkins. 2014. Echinococcus multilocularis and E. Canadensis in Wolves from Western Canada. Parasitology 141: 159–63. https://doi.org/10.1017/S0031182013001716.

Schuster, RK., and Shimalov VV. (2017). A Comparative Study of Helminths of Raccoon Dogs (*Nyctereutes procynoides*) and Red Foxes (*Vulpes vulpes*) Sharing the Same Territory. Journal of Tropical Disease. 7(12), 708-174. <https://doi.org/10.12980/apjtd.7.2017D7-259>.

Sikó, Sandor B., Peter Deplazes, C. Ceica, C. S. Tivadar, I. Bogolin, S. Popescu, and V. Cozma. 2011. Echinococcus multilocularis in South-Eastern Europe (Romania). Parasitology Research 108 (5): 1093–97. <https://doi.org/10.1007/s00436-010-2150-1>.

Stien, A., Voutilainen, L., Haukisalmi, V., Fuglei, E., Mork, T., Yoccoz, N., Ims, R., and Henttonen, H. (2010). Intestinal Parasites of the Arctic Fox in Relation to the Abundance and Distribution of Intermediate Hosts. Parasitology. 137(1), 149-157. https://doi.org/10.1017/S0031182009990953.

Szostakowska, B., Lass, A., Kostyra, K., Pietkiewicz, H., and Myjak, P. (2014). First Finding of *Echinococcus multilocularis* DNA in Soil: Preliminary Survey in Varmia-Masuria Province, Northeast Poland. Veterinary Parasitology. 2013(102), 73-79. <https://doi.org/10.1016/j.vetpar.2014.02.028>.

Takahashi, K., Uraguchi, K., Hatakeyama, H., Giraudoux, P., and Romig, T. (2013). Efficacy of Anthelmintic Baiting of Foxes against *Echinococcus multilocularis* in Northern Japan. Veterinary Parasitology. 198(1–2), 122–26. <https://doi.org/10.1016/j.vetpar.2013.08.006>.

Tolnai, Z., Széll, Z. and Sréter, T. (2013). Environmental Determinants of the Spatial Distribution of *Echinococcus multilocularis* in Hungary. Veterinary Parasitology. 198(3–4): 292–97. <https://doi.org/10.1016/j.vetpar.2013.09.004>.

Torgerson, PR., Rosenheim, K., Tanner, I., Ziadinov, I., Grimm, F., Brunner, M., Shaiken, S., Shaikenov, B., Rysmukhambetova, A., and Deplazes, P. (2009). Echinococcosis, Toxocarosis and Toxoplasmosis Screening in a Rural Community in Eastern Kazakhstan. Tropical Medicine and International Health, 14(3), 341-348. <https://doi.org/10.1111/j.1365-3156.2009.02229.x>.

Umhang, G., Woronoff-Rhen, N., Combes, B., and Boué, F. (2011). Segmental Sedimentation and Counting Technique (SSCT): An Adaptable Method for Qualitative Diagnosis of *Echinococcus multilocularis* in Fox Intestines. Experimental Parasitology. 128 (1): 57–60. <https://doi.org/10.1016/j.exppara.2011.01.004>.

Umhang, G., Raton, V., Comte, S., Hormaz, V., Boucher, JM., Combes, B. and Boué, F. (2012). *Echinococcus multilocularis* in Dogs from Two French Endemic Areas: No Evidence of Infection but Hazardous Deworming Practices. Veterinary Parasitology. 188 (3–4): 301–5. <https://doi.org/10.1016/j.vetpar.2012.03.024>.

Umhang, G., Comte, S., Raton, V., Hormaz, V., Boucher, JM., Favier, S., Combes, B., and Boue, F. (2014). *Echinococcus multilocularis* Infections in Dogs from Urban and Peri-Urban Areas in France. Parasitology Research. 113(6), 2219-2122 . <https://doi.org/10.1007/s00436-014-3875-z>.

Umhang, G., Lahoreau, J., Hormaz, V., Boucher, JM., Guenon, A., Montange, D., Grenouillet, F., and Boue, F. (2016). Surveillance and Management of *Echinococcus multilocularis* in a Wildlife Park. Parasitology International. 65(3), 245-250. <https://doi.org/10.1016/j.parint.2016.01.008>.

Villeneuve, A., Polley, L., Jenkins, EJ., Schurer, JM., Gilleard, J., Kutz, SJ., Conboy, G., Benoit, D., Seewald, W., and Gagne, F. (2015). Parasite Prevalence in Fecal Samples from Shelter Dogs and Cats across the Canadian Provinces. Parasites & Vectors. 8, 281.

王桂芝 冯晓辉 初向东 尔西丁 阿米娜 周吉霞 王巧 贺金华 温浩（2009）. 年新疆和布克赛尔蒙古自治县棘球蚴病现况调查. 中国地方病学杂志,28: 214-217

王桂芝 冯晓辉 初向东 尔西丁 阿米娜 周吉霞 王巧 贺金华 温浩（2009). 年新疆和布克赛尔蒙古自治县棘球蚴病现况调查. 中国地方病学杂志,28: 214-217. [Wang G, Feng X, Chu X, Amina E, Zhou J, Wang Q, He J, Wen H. Epidemic status of alveolar echinococcosis in Tibetan children in south Qinghai Province: Epidemiological study on human echinococcosis in Hobukesar Mongolian Autonomous County of Xinjiang. Chin J Endemiol. 2009; 28:214-217.]

Wang, Q., Raoul, F., Budke, C., Craig, P., Xiao, YF., Vuitton, D., Campos-Ponce, M., Qiu, DC., Pleydell, D., and Giraudoux, P. (2010). Grass Height and Transmission Ecology of *Echinococcus multilocularis* in Tibetan Communities, China. Chinese Medical Journal. 123(1), 61-67. <https://doi.org/10.3760/cma.j.issn.0366-6999.2010.01.011>.

Yang, YR., Craig, PS., Vuitton, DA., Williams, GM., Sun, T., Liu, TX., Boufana, B. *et al*. 2008. Serological Prevalence of Echinococcosis and Risk Factors for Infection among Children in Rural Communities of Southern Ningxia, China. Tropical Medicine and International Health. 13(8), 1086-1094. <https://doi.org/10.1111/j.1365-3156.2008.02101.x>.

Yu, SH., Wang, H., Wu, XH., Ma, X., Liu, PY., Liu, YF., Zhao, YM., Morishima, Y. and Kawanaka, M. (2008). Cystic and Alveolar Echinococcosis: An Epidemiological Survey in a Tibetan Population in Southeast Qinghai, China. Japanese Journal of Infectious Disease. 61(3), 242-246.

赵玉敏. 青藏高原东段（甘南藏族自治州）包虫病的流行病学研究[D]. 甘肃农业大学, 2008. [Zhao, Y. Echinococcosis on the Eastern QingHai Tibet Plateau (Gannan Tibetan Autonomous Prefecture). 2008. Gansu Agricultural University, Lanzhou, Gansu, China.]

Ziadinov, I., Mathis, A., Trachsel, D., Rysmukhambetova, A., Abdyjaparov, TA., Kuttubaev, OT., Deplazes, P., and Torgerson, PR. (2008). Canine Echinococcosis in Kyrgyzstan: Using Prevalence Data Adjusted for Measurement Error to Develop Transmission Dynamics Models. International Journal for Parasitology. 38(10), 1179–1190.

Ziadinov, I., Deplazes, P., Mathis, A., Mutunova, B., Abdykerimov, K., Nurgaziev, R., and Torgerson, PR. (2010). Frequency Distribution of *Echinococcus multilocularis* and Other Helminths of Foxes in Kyrgyzstan. Veterinary Parasitology. 171(3-4), 286-292. <https://doi.org/10.1016/j.vetpar.2010.04.006>.
